# Rarity of microbial species: In search of reliable associations

**DOI:** 10.1371/journal.pone.0200458

**Published:** 2019-03-15

**Authors:** Arnaud Cougoul, Xavier Bailly, Gwenaël Vourc’h, Patrick Gasqui

**Affiliations:** UMR Epidemiology of Animal and Zoonotic Diseases, Université Clermont Auvergne, INRA, VetAgro Sup, Saint-Genès-Champanelle, France; Wageningen University, NETHERLANDS

## Abstract

The role of microbial interactions in defining the properties of microbiota is a topic of key interest in microbial ecology. Microbiota contain hundreds to thousands of operational taxonomic units (OTUs), most of them rare. This feature of community structure can lead to methodological difficulties: simulations have shown that methods for detecting pairwise associations between OTUs, which presumably reflect interactions, yield problematic results. The performance of association detection tools is impaired when there is a high proportion of zeros in OTU tables. Our goal was to understand the impact of OTU rarity on the detection of associations. We explored the utility of common statistics for testing associations; the sensitivity of alternative association measures; and the performance of network inference tools. We found that a large proportion of pairwise associations, especially negative associations, cannot be reliably tested. This constraint could hamper the identification of candidate biological agents that could be used to control rare pathogens. Identifying testable associations could serve as an objective method for filtering datasets in lieu of current empirical approaches. This trimming strategy could significantly reduce the computational time needed to infer networks and network inference quality. Different possibilities for improving the analysis of associations within microbiota are discussed.

## Introduction

Microbiota play key roles in ecosystem processes, from eukaryote physiology [1] to global biogeochemical cycles [2]. Research often focuses on comparing microbiota found in similar environments to identify the major forces shaping their structure [3] and function [4]. Microbial interactions are probably one such force [5, 6].

The most common technique for describing microbiota is 16S rRNA sequencing [7]. Association network analysis is then often employed to characterize potential microbial interactions [8]. Such analyses require identifying pairwise associations between the occurrence or abundance of bacterial operational taxonomic units (OTUs) [9]. However, microbiota frequently contain hundreds to thousands of OTUs, most of them rare [10–12]. Consequently, a typical matrix describing the abundance of OTUs among similar microbiota will include a high proportion of zeros. Simulations have illustrated that an excess of zeros impairs the efficiency of association network analysis [13, 14]. To avoid this problem, rare OTUs are filtered out beforehand. Current trimming procedures are empirical in nature and restrictive. They may rely on OTU prevalence [13, 15], mean abundance [16], or diversity [17]. Moreover, simulations have found that association network analyses more efficiently detect negative relationships (i.e., amensal, competitive) than positive relationships (i.e., mutualistic, commensal) [13]. It is not yet clear whether this result is due to the distribution of OTU prevalence.

Precisely defining the conditions under which positive and negative associations can be reliably tested should improve current research on microbial interactions. This approach could help design studies that have adequate statistical power; identify potential paths for improving data analysis; and, accounting for its constraints, clarify the interpretation of association network analyses.

Below, we analyzed the effect of low OTU prevalence, a common pattern in real microbiota, on association measures calculated from occurrence data and read abundance data. More specifically, we theoretically and empirically calculated the extrema of common prevalence-based association measures. These extrema were used to define which OTU associations could be reliably tested. We investigated whether alternative association measures and cutting-edge association network analysis tools were also affected by low OTU prevalence. This methodological strategy allowed us to (i) define the extent to which prevalence and sample size affect the results of microbiota association analyses; (ii) demonstrate that negative associations cannot be captured in most cases; and (iii) show that there is little added value obtained from analyzing abundance data as compared to occurrence data. We discuss our findings in the context of current analytical procedures and tools with a view to proposing potential solutions to the issues we identified.

## Materials and methods

Methods for detecting associations among microbes have progressed rapidly as the to obtain microbiota data has become more widespread. Here, we determined how an excess of zeros affected classical correlation measures by examining the latter’s testability. We also considered alternative association measures and explored the relationship between method type and association network inference quality.

Prevalence affects the distribution of association statistics, which can lead to problems with the testability of correlation coefficients. For instance, a statistic’s minimum and/or maximum can fall within the expected confidence interval obtained from the classical distributions used to approximate expected values. This issue can arise with both occurrence data and abundance data.

### Model for occurrence data: Fisher test and Phi coefficient

First, we explored how to define testability when occurrence data are used. Co-occurrence networks are commonly reconstructed using the hypergeometric law that underlies Fisher’s exact test [9, 18, 19]. For fixed prevalence values, the probability of observing the minimum or maximum number of co-occurrences may be higher than the alpha level (traditionally set to 5%) [20, 21]. In such a case, neither negative nor positive associations, respectively, can be significantly detected. Limits on testability can be studied by enumerating all the possible combinations of associations based on prevalence (detailed in Part B.7 in S1 Appendix). The combinatorics that ensue from the hypergeometric law provide numerical solutions for determining association testability.

The Phi coefficient [22] can be used to establish equations for exploring association testability, which provide an analytical solution. The Phi coefficient *ϕ* is a measure of association between two binary variables *X*_*A*_ and *X*_*B*_.

$$\begin{array}{c} \phi =\sqrt{\frac{P_{11}-P_{A}\;P_{B}}{P_{A}\;\left(1-P_{A}\right)\;P_{B}\;\left(1-P_{B}\right)}}, \end{array}$$ (1)

where *P*_*A*_, *P*_*B*_ are the prevalence values for two OTUs, *X*_*A*_ and *X*_*B*_, and *P*_11_ is the prevalence of their co-occurrence. The prevalence of an OTU is

$$\begin{array}{c} \text{prevalence}=\frac{\text{number}\;\text{of}\;\text{non-zero}\;\text{samples}}{\text{total}\;\text{number}\;\text{of}\;\text{samples}}. \end{array}$$ (2)

The extrema of Phi [23] depend exclusively on *P*_*A*_ and *P*_*B*_ (S1 Fig and Part B in S1 Appendix).

$$\begin{array}{c} \begin{array}{c} \text{min}\left(\phi \right)=\text{max}(-\sqrt{\frac{P_{A}\;P_{B}}{\left(1-P_{A})(1-P_{B}\right)}},-\sqrt{\frac{\left(1-P_{A})(1-P_{B}\right)}{P_{A}\;P_{B}}})\\ \text{max}\left(\phi \right)=\text{min}(-\sqrt{\frac{P_{A}(1-P_{B})}{P_{B}(1-P_{A})}},-\sqrt{\frac{P_{B}(1-P_{A})}{P_{A}(1-P_{B})}}) \end{array} \end{array}$$ (3)

Under the null hypothesis (*H*_0_) that the occurrences of *X*_*A*_ and *X*_*B*_ are independent, Phi can be approached thanks to Pearson’s chi-squared test:

$$\begin{array}{c} \phi^{2}=\frac{\chi^{2}}{N}, \end{array}$$ (4)

where *N* is the total number of samples and *χ*^2^ is a chi-squared distribution with one degree of freedom [24]. This latter distribution is thus used to build a confidence interval with which to test departure from the null hypothesis. Furthermore, we can describe cases where it would be impossible to reliably test associations based on this confidence interval because the genuine minimum and/or maximum of *ϕ* fall within the confidence interval.

### Model for read abundance data: Pearson and Spearman correlations

Second, we explored how to define testability when read abundance data are used. We first employed the Pearson correlation coefficient [25], which is a measure of association between two continuous variables, *X*_*A*_ and *X*_*B*_.

$$\begin{array}{c} r=\frac{E\left(X_{A}\;X_{B}\right)-E\left(X_{A}\right)\;E\left(X_{B}\right)}{\sigma_{X_{A}}\;\sigma_{X_{B}}} \end{array}$$ (5)

We demonstrated that the minimum of the Pearson correlation coefficient depends only on OTU prevalence (see the proof in Part C in S1 Appendix and the illustration in S2 Fig).

$$\begin{array}{c} \text{min}\left(r\right)=-\sqrt{\frac{P_{A}\;P_{B}}{\left(1-P_{A})(1-P_{B}\right)}}>-1,\;\text{if}\;P_{A}+P_{B}<1 \end{array}$$ (6)

We can then define a confidence interval based on the following assumption: if *X*_*A*_ and *X*_*B*_ follow two uncorrelated normal distributions,

$$\begin{array}{c} r=\frac{t}{\sqrt{N-2+t^{2}}} \end{array}$$ (7)

where *t* has a Student’s *t*-distribution with degrees of freedom *N* − 2.

We demonstrated that the result for the correlation minimum (Eq (6) is identical for the Spearman correlation approach (Part C.7 in S1 Appendix). The Spearman correlation is the Pearson correlation applied to the ranks of *X*_*A*_ and *X*_*B*_. The Spearman correlation coefficient follows the same expected distribution described by Eq (7) when *X*_*A*_ and *X*_*B*_ are independent. This fact makes it possible to relax the assumption of normality of the Pearson correlation test, a hypothesis not respected in the analysis of the microbiota data.

To estimate the proportion of unreliable tests, we considered two distributions for OTU prevalence: (i) a uniform law, to study the influence of sample size *N* and prevalence *P*_*A*_, *P*_*B*_ and (ii) a truncated power law, to take into account the real structure of microbiota data. We also compared the results for the testability limits for the two types of data and highlighted a correlation between the two associated measures.

### Simulated responses of association measures

We found that, theoretically, OTU prevalence has an impact on the observable minimum Pearson and Spearman correlation coefficients. We therefore explored the behavior of alternative association measures. We analyzed the relationship between OTU prevalence and the values of five measures used to infer association networks: Pearson and Spearman correlation coefficients, Bray Curtis dissimilarity, mutual information, and the maximal information coefficient (MIC) [9, 26]. Bray Curtis dissimilarity is an ecological statistic that we employed here to quantify compositional dissimilarity between OTUs. Mutual information and the MIC are two measures that were developed from information theory. Both are used to capture nonlinear or non-monotonic relationships. We generated two correlated variables to analyze the responses of the association measures. The zero-inflated negative binomial (ZINB) distribution appears to best fit microbiota data [27, 28]. We generated a bivariate normal sample of size *N* = 50 and simulated three correlation levels: a negative correlation (*r* = 1), a positive correlation (*r* = 1), and a null correlation (*r* = 0), which served as a reference. The copula theory allows normally distributed data to be marginally transformed into ZINB-distributed data [29]. OTU prevalence was modeled using the probability of structural zeros. For the ZINB distribution, dispersion was 0.5, and the mean was 1000. This situation corresponded to two OTUs of high abundance. Prevalence values ranged from 0.05 to 0.95 in 0.05 steps. We calculated the value of each association measure for all possible pairs of prevalence. We conducted 100 simulations and retained the median value for each prevalence pair.

### Association network analysis tools

We studied the relationship between OTU prevalence and the quality of inference provided by association network analysis tools. Three inference tools were studied: CoNet [30], SPIEC-EASI [15], and SparCC [16]. We simulated datasets containing 50 samples and 100 OTUs. The data followed a multivariate normal distribution and contained with 100 known associations, of which half were positive and half were negative. From the adjacency matrix, we calculated a correlation matrix where the target matrix condition was 100, as described in [15]. Using the copula theory, we then transformed the normally distributed data into ZINB-distributed data [29]. Prevalence was modeled using the probability of structural zeros. All the OTUs had the same prevalence, which was the variable study parameter. For the ZINB distribution, dispersion was 0.5, and the mean was 1000. Finally, we used the different tools to infer the association network and measured tool ability to pick up on positive or negative associations. We independently examined the proper classification of negative associations and positive associations. Inference quality was assessed based on the area under the ROC curve (AUC) and the area under the precision-recall curve (AUPRC) [31].

### Data filtering before association network inference

We analyzed the effect of data filtering methods on network inference quality. We simulated datasets containing 300 samples and 300 OTUs following a ZINB distribution, as described in the previous paragraph. The datasets contained 1000 associations, half positive and half negative. As above, the target matrix condition was 100. OTU prevalence followed a power law distribution where *k* = −1.5. Minimum prevalence was 5/300 to avoid simulating a situation in which OTUs were missing from all 300 samples, which would not be taken into account in network inference. For the ZINB distribution, dispersion was 0.5, and the mean was 1000. We implemented data filtering in CoNet and SPIEC-EASI (S2 File). For CoNet, we did not compute the p-values of the problematic pairs we identified. For SPIEC-EASI, after normalizing the data with the centered log-ratio (clr) transformation [32], we assigned a zero weight to the problematic pairs during the graphical lasso estimation [33], which corresponded to a strong regularization parameter for these pairs. SparCC’s algorithm did not allow problematic pairs to be filtered. To generate benchmarks for data filtering, we inferred association networks under three different conditions: (i) for all OTU pairs; (ii) for fully testable pairs only (i.e., after removing problematic pairs; alpha level of 5%); and (iii) for OTUs that had been filtered based on a prevalence threshold. In this latter case, the goal was to be able to compare the results of filter based on testability with those obtained using a conventional filter based on prevalence. To do this, we removed enough low prevalence OTUs to have at least the same number of filtered pairs as in (ii). We performed 20 simulations of each. We then measured the AUC values associated with network inference. Inference quality was based only on the associations that remained after filtering.

## Results

### Testability given a uniform prevalence distribution

**Fig 1:**
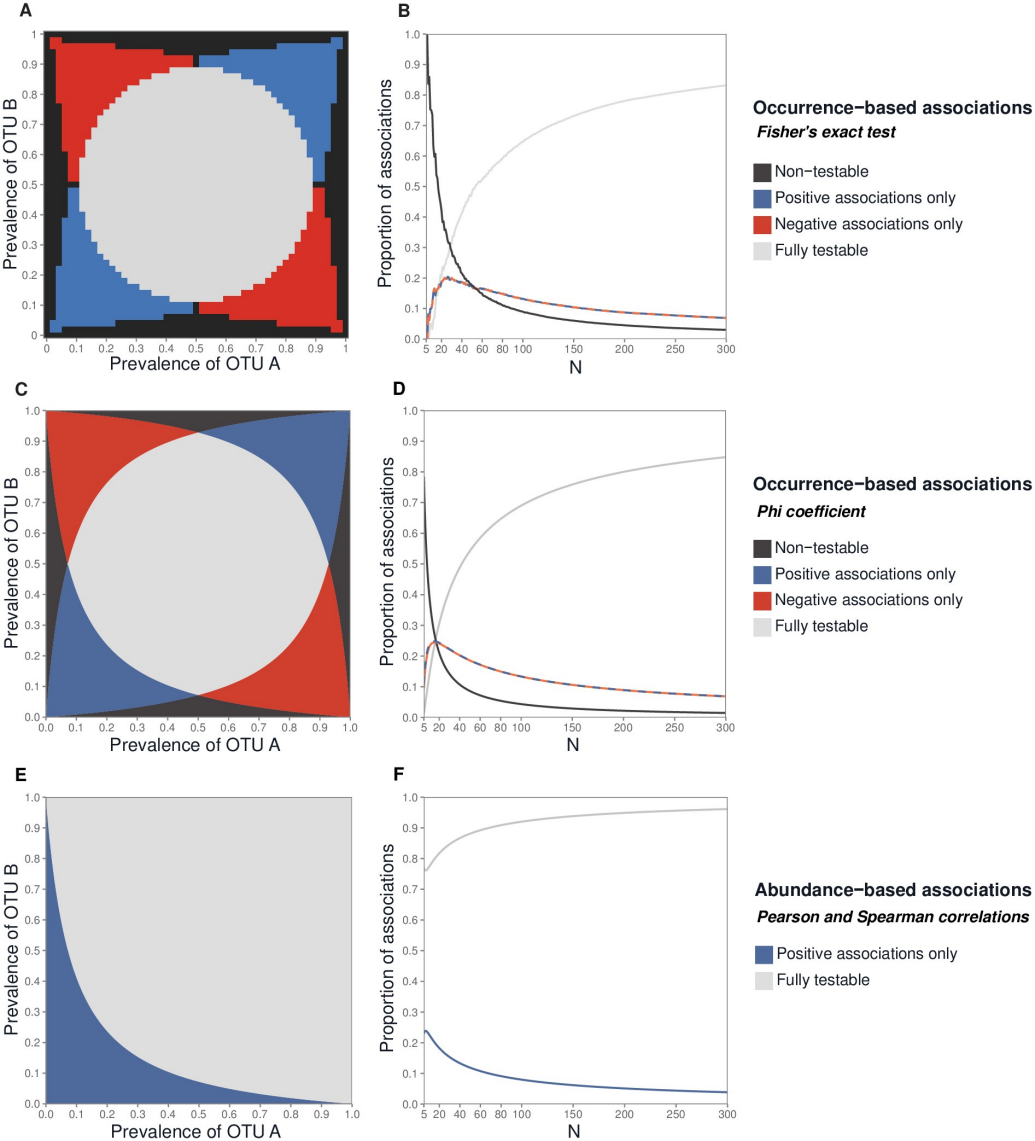
Testability of pairwise associations for the occurrence data and for the read abundance data. For the occurrence data: the testability zones defined by OTU prevalence for the Fisher’s exact test (**A**), and the proportion of testable associations as a function of *N* assuming prevalence follows a uniform distribution (**B**). Testability zones defined by OTU prevalence for the Phi coefficient (**C**), and the proportion of testable associations as a function of *N* assuming prevalence follows a uniform distribution (**D**). For the read abundance data: testability zones defined by OTU prevalence for the Pearson and Spearman correlation coefficients (**E**), and the proportion of testable associations as a function of *N* assuming prevalence follows a uniform distribution (**F**). The alpha level for the tests was 5%. For (A), (C) and (E), *N* = 50.

When occurrence data were used, four inequations (Eqs (7-10) in S1 Appendix) defined reliable tests based on the chi-squared distribution and OTU prevalence (Fig 1C). The proportion of non-testable associations (i.e., neither positive nor negative correlations could ever be significant) rapidly fell as *N* increased (Fig 1D). The proportion of associations with partial testability (i.e., either only positive or negative correlations could ever be significant) never exceeded 0.25 (Fig 1D). When *N* = 300, the proportion of fully testable associations (both positive and negative correlations could be significant) exceeded 0.80 (Fig 1D). We showed numerically that there was consistency between the proportion of Fisher’s exact tests affected by prevalence and the analytical results (Fig 1A and 1B). There were slightly more non-testable associations when Fisher’s exact test was used, as compared to the Phi coefficient, and slightly fewer associations with partial testability.

When read abundance data were used, some negative correlations were not testable based on the Student’s distribution (Eq (33) in S1 Appendix and Fig 1E). This problem became less pronounced as *N* increased, and the proportion of testable associations reached 0.95 at *N* = 300 (Fig 1F).

### Testability given realistic community structure

**Fig 2:**
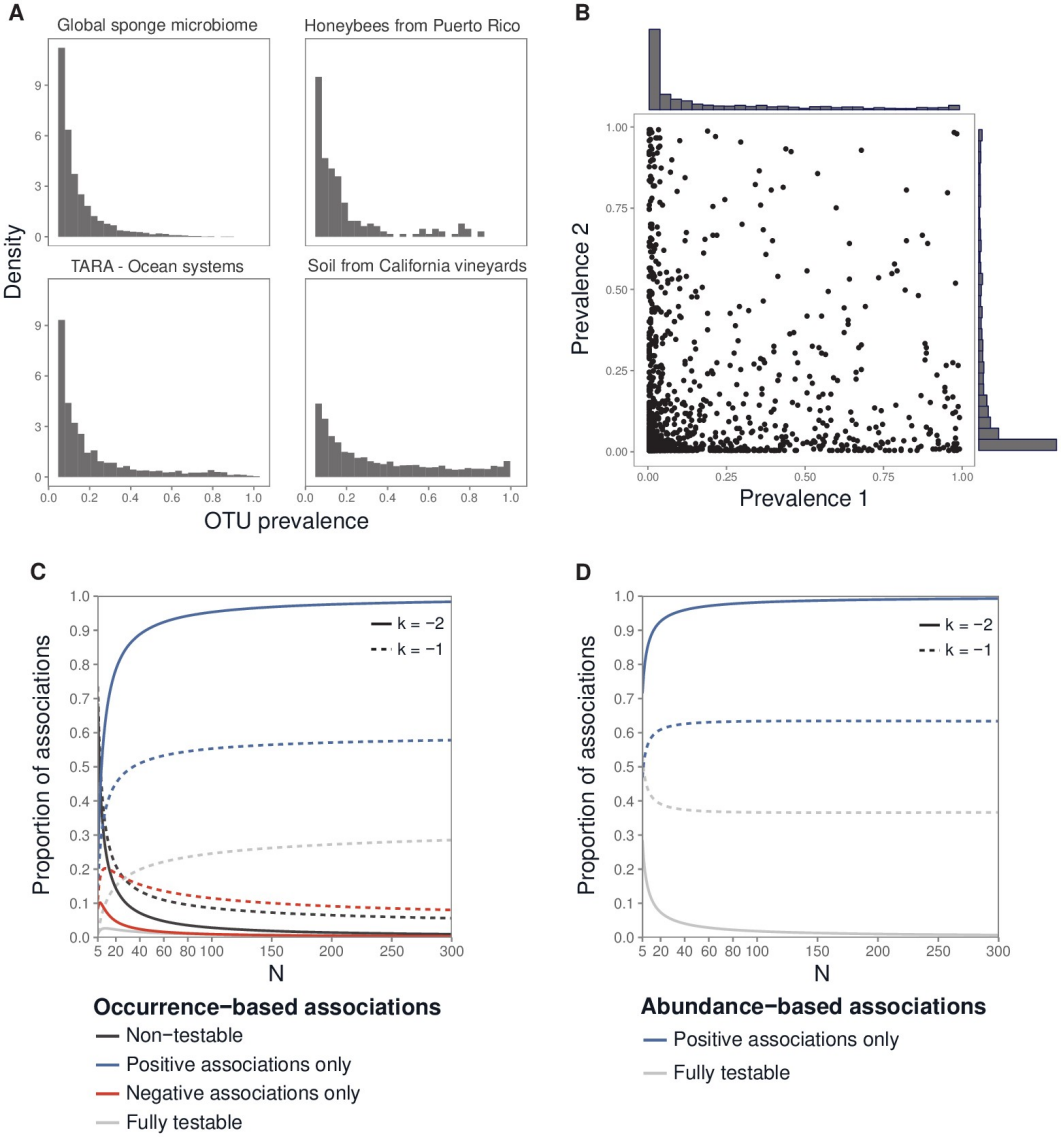
Association testability under realistic conditions of microbial community structure. (**A**) Histograms of OTU prevalence in several microbiota characterized by 16S rRNA sequencing. Data were taken from the Qiita database [34] and the TARA Ocean Project [35]. The microbiota are described elsewhere (Part E in S1 Appendix). To better illustrate the skewed distributions, only prevalence values of greater than 5% were included. (**B**) Distribution of all pairs of OTU prevalences from microbiota data for soil from California vineyards. Each point represent a pair of OTU prevalences. Proportion of testable associations as a function of *N* when *k* = −2 or −1 for the occurrence data (**C**) and the read abundance data (**D**).

Prevalence distributions are highly unbalanced in microbiota because of the large number of rare OTUs (Fig 2A). Accordingly, we modeled OTU prevalence using a truncated power law distribution; the latter reflects observed community structure (Part E in S1 Appendix and S3 Fig). OTU prevalence was fitted according to a truncated power law, with *k* ranging from −2 to −1: the smaller the *k*, the higher the proportion of rare species. The use of such a distribution means that, for most OTU pairs, both OTUs had a low prevalence (Fig 2B).

For the occurrence data, there was thus a large proportion of associations for which negative correlations could never be significant (> 0.50 for *k* = −1, > 0.90 for *k* = −2); this proportion increased as *N* increased (Fig 2C). This counter-intuitive result is due to the accumulation of rare OTUs as N increases under the power law assumption. Fewer than 10% of associations were non-testable when *N* was greater than 50 (Fig 2D).

For the read abundance data, when *N* = 100, a large proportion of negative correlations were non-testable when *k* = −1 (proportion: 0.60) and *k* = −2 (proportion: 0.95) (Fig 2D).

### Comparison between the two data types

**Fig 3:**
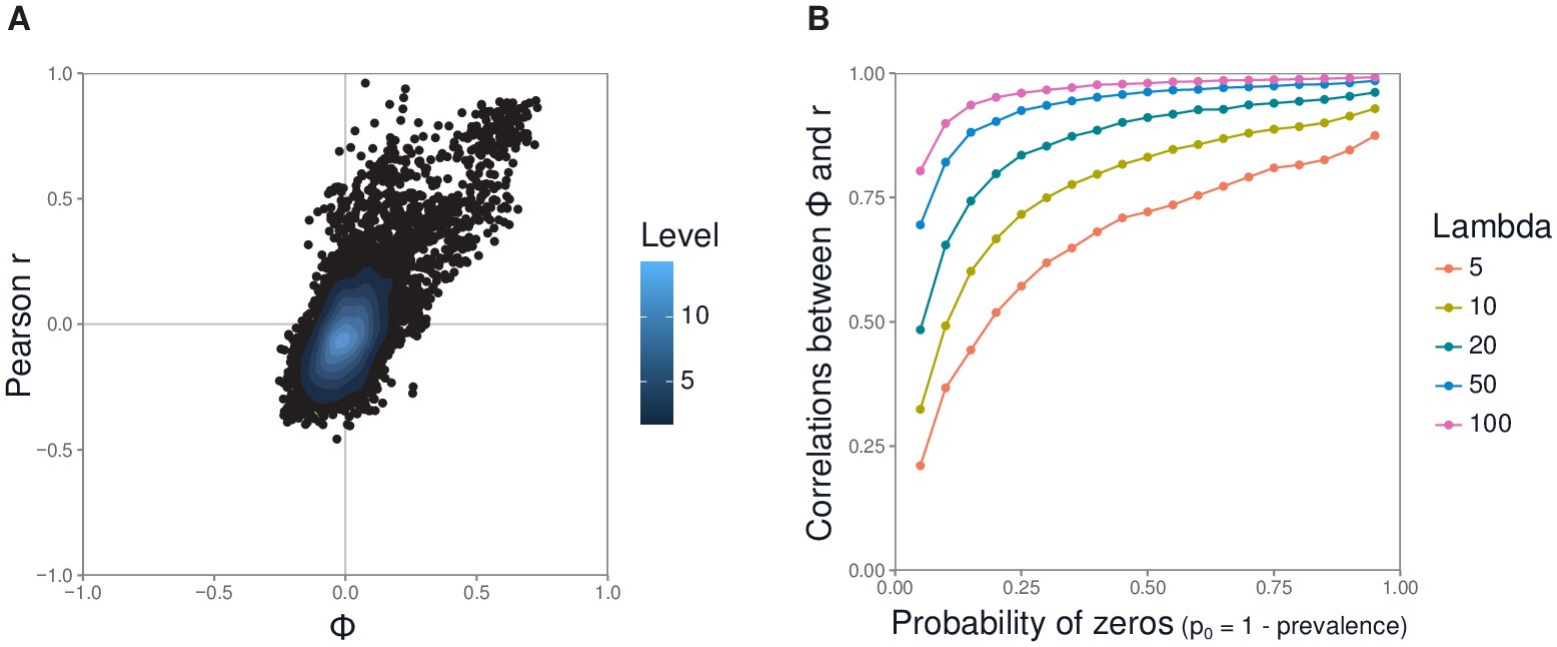
Correlation between the Phi coefficient and the Pearson coefficient. (**A**) Correlation in honeybees microbiota data (Part E in S1 Appendix). Each point corresponds to the association coefficients for an OTU pair. Read abundance data were normalized using clr. (**B**) Correlation computed from simulations of OTU abundances modeled using a zero-inflated Poisson (ZIP) distribution (Part D.2 in S1 Appendix). The parameters are the probability of structural zeros, *p*_0_, and the value of the Poisson parameter, λ. In biological terms, the probability of structural zeros corresponds to the prevalence (prevalence = 1 − *p*_0_), and the Poisson parameter corresponds to read abundance. For each pair of *p*_0_ and λ values, we generated 100 samples of two hypothetical OTUs, *X*_*A*_ and *X*_*B*_, whose abundances followed a ZIP distribution with those parameter values. We then calculated *ϕ* and *r* for the samples. The correlation between the two coefficients was assessed by repeating this process 10^5^ times.

We compared the association statistics for both data types under conditions of low OTU prevalence such as those observed in actual microbiota data (Part D in S1 Appendix). A formal decomposition of variance and covariance illustrates the structural relationship of the correlation coefficients calculated from the occurrence data and the read abundance data (Eq (2), Part A in S1 Appendix). The observed values of the Phi coefficient *ϕ* and the Pearson correlation coefficient *r* for OTU pairs in microbial datasets (Fig 3A) illustrated that the minimum of the statistics is particularly affected as explained above. Furthermore a correlation was observed between the two measures for real microbiota datasets (cor = 0.78 and *R*^2^ = 0.62 for honeybees microbiota data, Fig 3A). Simulations allowed us to delve deeper into the expected correlation between the two measures. The association tests that can be performed using occurrence data versus read abundance data tend to be similar, and prevalence influences association testability in the same way. More specifically, association measures for the two data types become correlated as prevalence decreases (Fig 3B).

### Impact of OTU prevalence on other association measures

**Fig 4:**
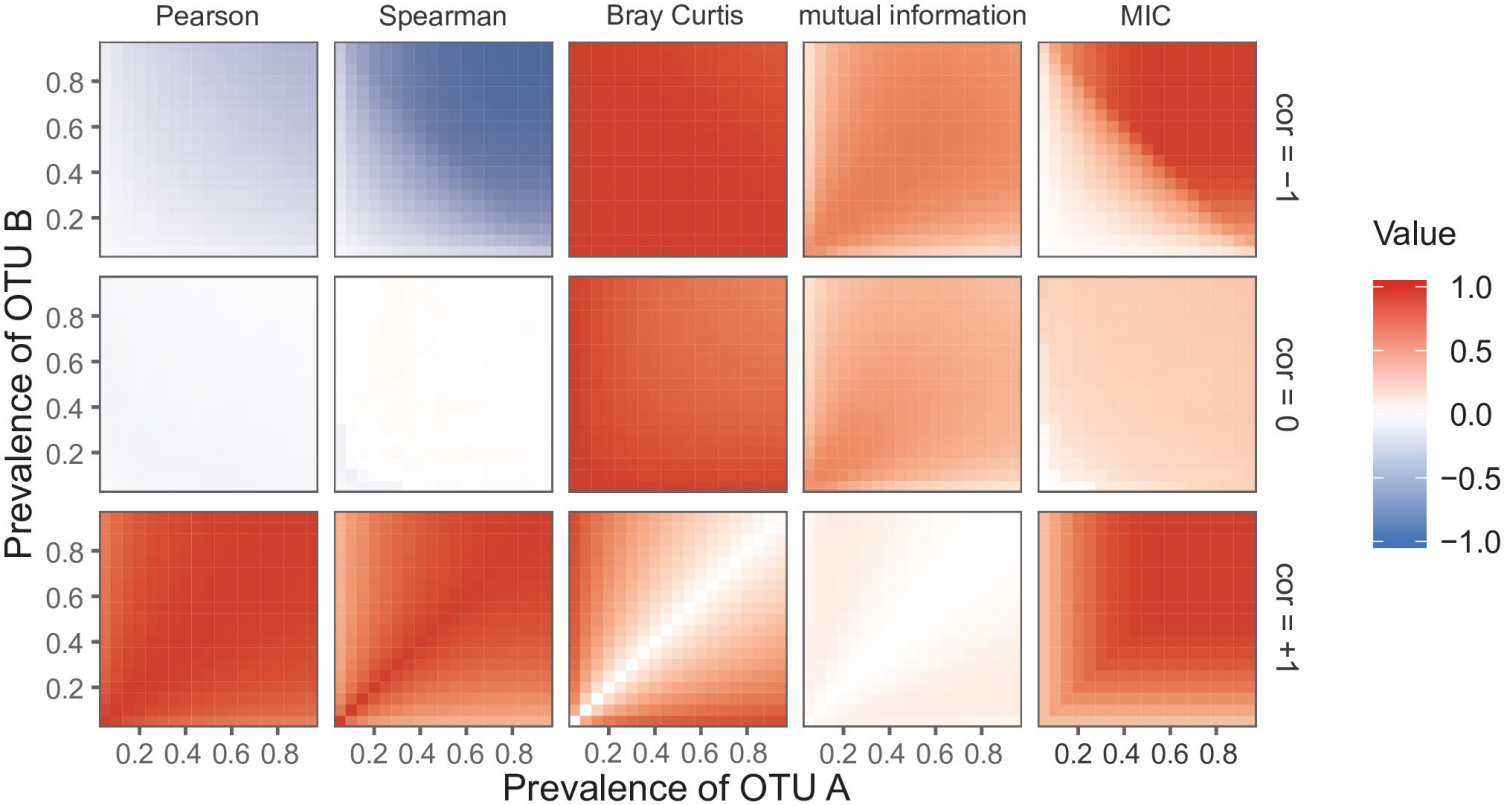
Relationship between OTU prevalence and the responses of five association measures for a simulated dataset. Two zero-inflated negative binomial (ZINB) distributions (*N* = 50, *μ* = 1000, *θ* = 0.5, *p*_0_ = 1 − prevalence) were created using all pair of prevalences from 0.05 to 0.95 (steps of 0.05) and for three correlation levels. For the graphs, the correlation level is −1 in the first row, 0 in the second row, and +1 in the third row. The five association measures are represented in different columns. A total of 100 simulations were performed, and the median values were plotted.

We studied the relationship between OTU prevalence and the responses of five common association measures (Fig 4) using simulated data. There were differences in the abilities of the measures to capture negative associations. The Pearson correlation coefficient did a poor job of picking up on negative associations. The Spearman correlation coefficient did better: it was able to pick up on negative associations. OTU prevalence had a strong effect on the Spearman correlation coefficient, as noted above. Bray Curtis dissimilarity and mutual information did a poor job of capturing negative associations: their responses for the dataset containing the associations were the same as their responses for the null dataset. The MIC responded well, especially when prevalence was high. The Spearman correlation coefficient and the MIC were the only measures that could properly capture negative correlations, but they were nonetheless affected by low prevalence.

In the case of the positive associations, all five measures showed a greater degree of sensitivity. However, OTU prevalence still exerted an influence, even if it was less dramatic than for negative associations. For the negative associations, measures were altered when the sum of the two prevalences decreased, along the first bisector. For the positive associations, measures were affected when one of prevalences decreased, along the prevalence axes. Consequently, the mechanisms that limit the ability to measure positive associations are different from those tied to negative associations.

### Impact of prevalence on network inference quality

**Fig 5:**
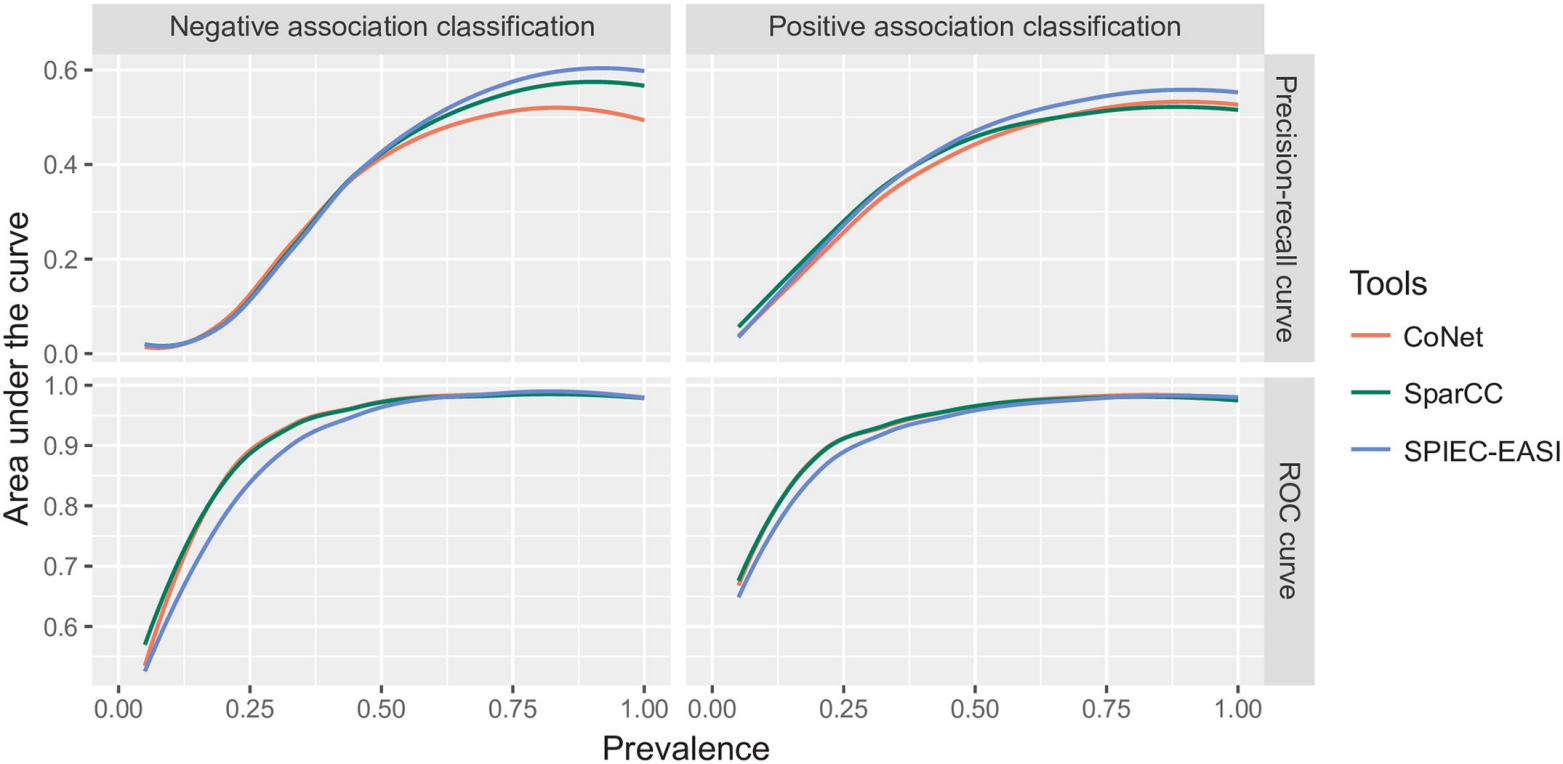
Performance of three association network analysis tools as a function of OTU prevalence. Datasets of 100 OTUs were generated using a ZINB distribution (*N* = 50, *μ* = 1000, *θ* = 0.5, *p*_0_ = 1 − prevalence). A covariance structure was imposed on the datasets-there were 100 associations, of which half were positive and half were negative. All OTUs had the same prevalence, which varied from 0.05 to 1 in 20 log steps. For each prevalence value, 20 simulations were performed. The plots show the means of a LOESS regression. The left-hand graphs represent the classification of the negative associations, and the right-hand graphs represent the classification of the positive associations. The top and bottom graphs show the AUPRC and AUC values, respectively.

We compared the ability of three recently developed tools to infer association networks within simulated microbiota data: all three had difficulties detecting associations when faced with a high proportion of zeros (i.e., low OTU prevalence; Fig 5). Positive associations were easier to detect, but low prevalence still had an effect. Examining the characteristics of the ROC curves, limitations occurred at a prevalence level of 0.2. When paired OTUs had prevalences below this level, they fell completely within a zone of partial testability, where only positive associations could be tested (compare with Fig 1E).

### Impact of data filtering on association network inference quality

**Fig 6:**
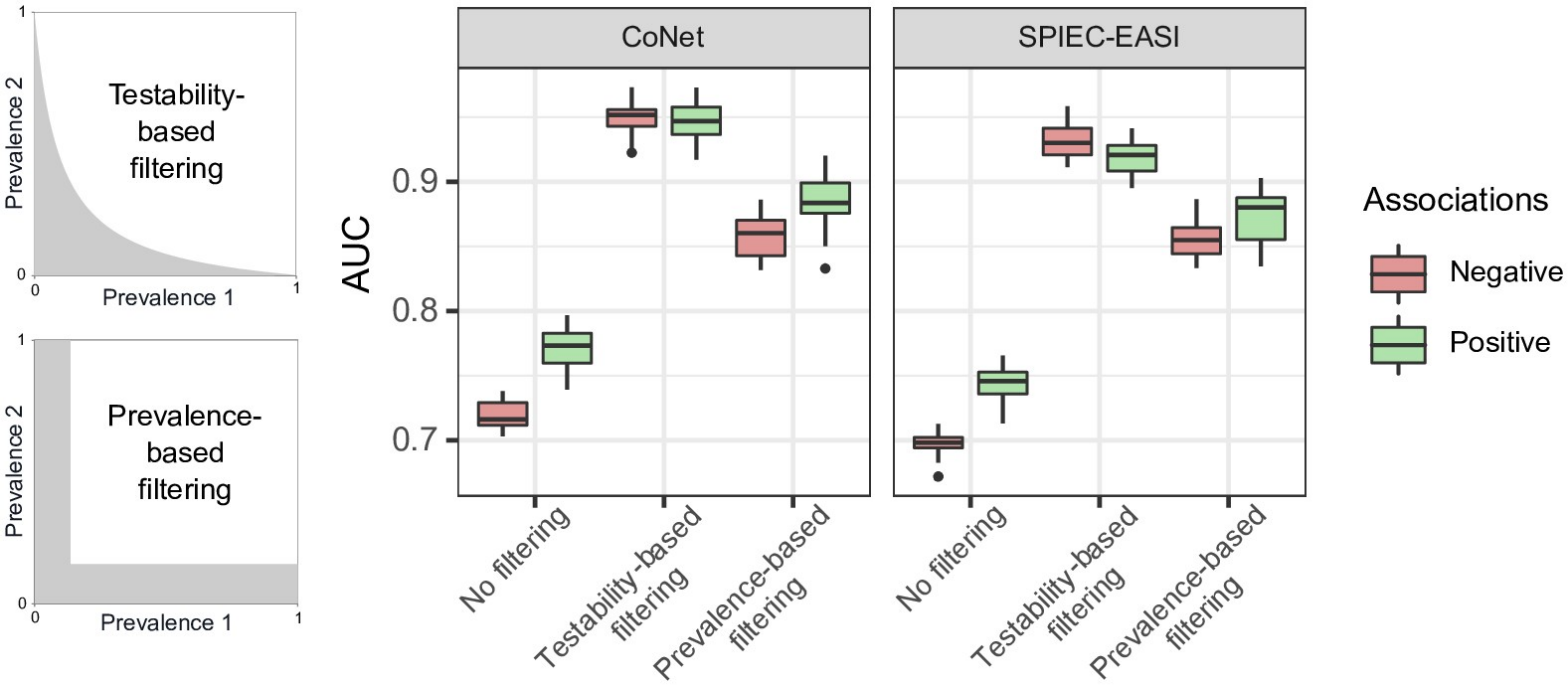
Impact of data filtering on association network inference quality. Performance of CoNet and SPIEC-EASI depending on data filtering methods: no filtering, testability-based filtering, and prevalence-based filtering. In testability-based filtering, problematic associations were removed (alpha level of 5%). In prevalence-based filtering, the lowest-prevalence OTUs were removed to obtain the same number of filtered associations as for testability-based filtering. Datasets of 300 OTUs were generated using ZINB distributions (*N* = 300, *μ* = 1000, *θ* = 0.5, *p*_0_ = 1 − prevalence). Prevalences were simulated using a power law distribution where *k* = −1.5. A covariance structure was imposed on the datasets: there were 1000 associations, half positive, half negative. The target matrix condition was 100. A total of 20 simulations were performed to obtain the boxplots of the areas under the ROC curve.

We analyzed the effect of filtering data on the quality of association network inference (Fig 6) using simulated data. In our dataset, problematic pairs (at an alpha level of 5%) represented, on average, 70% of the total number of associations. During prevalence-based filtering, we removed the less prevalent OTUs, with a view to eliminating the same proportion of associations as in testability-based filtering.

The results obtained with CoNet and SPIEC-EASI were quite similar. When the data were unfiltered, negative associations were less well recovered than were positive associations, as mentioned previously. Under these conditions, the AUC values were below 0.8. When the data were filtered, the quality of inference improved. When the data were filtered by testability, the AUC values for both negative and positive associations were greater than 0.9. Furthermore, the AUC values for negative associations were the same as the AUC values for positive associations (and were even higher when SPIEC-EASI was used). When prevalence-based filtering was used, the AUC values were lower. For our simulated dataset, testability-based filtering thus yielded better results than the more common, prevalence-based filtering procedure.

Network inference could be carried out significantly faster when the data were filtered. The mean calculation times for unfiltered, testability-filtered, and prevalence-filtered data were as follows: 122, 72, and 19 seconds, respectively, for SPIEC-EASI and 2041, 667, and 661 seconds, respectively, for CoNet.

## Discussion

We showed that it is impossible to reliably test a large proportion of the pairwise associations between OTUs in microbiota using classical association measures and common association network analysis tools. Indeed, in our simulations employing realistic community structure (i.e., most OTUs are rare), we discovered the following: (i) correlations, especially negative correlations, could not be tested for most associations using classical statistics; (ii) alternative association measures was also affected by low OTU prevalence; and (iii) cutting-edge network analysis tools also struggle when OTU prevalence is low. These findings clarify previous modeling results [13] and underscore a major analytical challenge in this domain. This issue cannot be solved via the use of statistics adapted to non-linear relationships, the permutation and bootstrap tests proposed by CoNet, or the clr transformation procedure employed by SPIEC-EASI. It also has important practical implications. For example, this constraint could hamper the identification of candidate biological agents that could be used to control rare pathogens.

We defined equations that can be used to quickly identify *a priori* whether OTU associations can be tested. Applying stringent standards (i.e., analyzing only fully testable associations) drastically reduced the number of tests required to infer an association network. We propose a way to implement this filtering strategy in CoNet and SPIEC-EASI: by assuming there is no association for problematic pairs in the correlation matrix of OTU abundances when an association network is being inferred. By limiting test number, the time needed for network inference was drastically reduced. We showed that identifying testable associations could serve as an alternative to current, empirical strategies for filtering microbiota datasets. Indeed, we found that inference quality may be better if data are filtered to remove problematic pairs of OTUs rather to remove low-prevalence OTUs.

We found that association testability tended to be similar for occurrence data and read abundance data. More specifically, association measures calculated using the two data types became correlated as prevalence decreased. This fact raises questions about the information that is actually being captured by current methods for quantifying OTU associations. These questions have both computational implications—it is unclear that current models are able to make the most of abundance data—and biological implications—the two data types could reveal the operation of different biological processes involved in interactions. Zero-inflated distributions can be used to explicitly model occurrence and abundance. They aim to differentiate structural zeros, due to OTU absence, from sampling zeros, due to limited sequencing depth. Since zeros can be ambiguous, presence-absence patterns likely change with sequencing depth. As a result, the minima and maxima of the Pearson correlation coefficient and the Phi coefficient will depend on this depth. Fitting OTU abundances using such distributions appears to be a promising solution for improving the inference of microbial associations [27, 36].

The low prevalence of OTUs in metagenomics datasets greatly limits our ability to carry out broad-scale analyses. Based on the results obtained in this study, we believe that advances in the discovery of microbial associations should be made by systematically integrating available information into the models being used. Initial attempts to develop statistical models that incorporate previous findings into metagenomics analyses have yielded promising results [37]. From a biological point of view, this approach would benefit from the development of a database dedicated to microbial interactions. Open and shared microbiota datasets, like those present on the Qiita collaborative platform (https://qiita.ucsd.edu), could be used to benchmark statistical models, and contributing to such databases could improve our knowledge of microbiota.

## Supporting information

S1 Fig Extrema of the Phi coefficient as a function of OTU prevalence. Minimum (**A**) and maximum (**B**) of the Phi coefficient as a function of prevalence. Computed from Eq (3).
(PDF)

S2 Fig Minimum of the Pearson correlation coefficient as a function of OTU prevalence. Minimum of the Pearson correlation coefficient *r* as a function of prevalence. Computed from Eq (6).
(PDF)

S3 Fig Prevalence structure of real microbial communities. (**A**) Histograms of OTU prevalence in several microbiota characterized by 16S rRNA sequencing. The microbiota are described in Part E in S1 Appendix. (**B**) Probability density function of the same prevalence data (log-log scale), which were fitted to a truncated power law distribution; the power law coefficient *k* was estimated by maximizing log-likelihood.
(PDF)

S4 Fig Proportion of testable associations as a function of the power law coefficient *k*. Proportion of testable associations as a function of *k* when *N* = 50, 100, or 300 for the occurrence data (**A**) and for the read abundance data (**B**).
(PDF)

S1 Appendix Supplementary material. 1. Notation and decomposition of variance and covariance. 2. Threshold method for binary data. 3. Threshold method for quantitative data. 4. Similarity of the Phi and Pearson correlation coefficients. 5. Distribution of OTU prevalence in real microbiota.
(PDF)

S1 File R code for carrying out testability-based data filtering. There are two functions, one for each data type: occurrence data and abundance data.
(R) Click here for additional data file.

S2 File Material used in the simulations. Code files, data files, and result files associated with each of the figures. Implementation of testability-based filtering in CoNet and via the graphical lasso method with clr transformation (SPIEC-EASI-like).
(ZIP)
