# Supplementary figures and images for: Rarity of microbial species: In search of reliable associations
Source: PLoS One. 2019 Mar 15;14(3):e0200458. doi: 10.1371/journal.pone.0200458 (PMC6420159; doi:10.1371/journal.pone.0200458)

**A**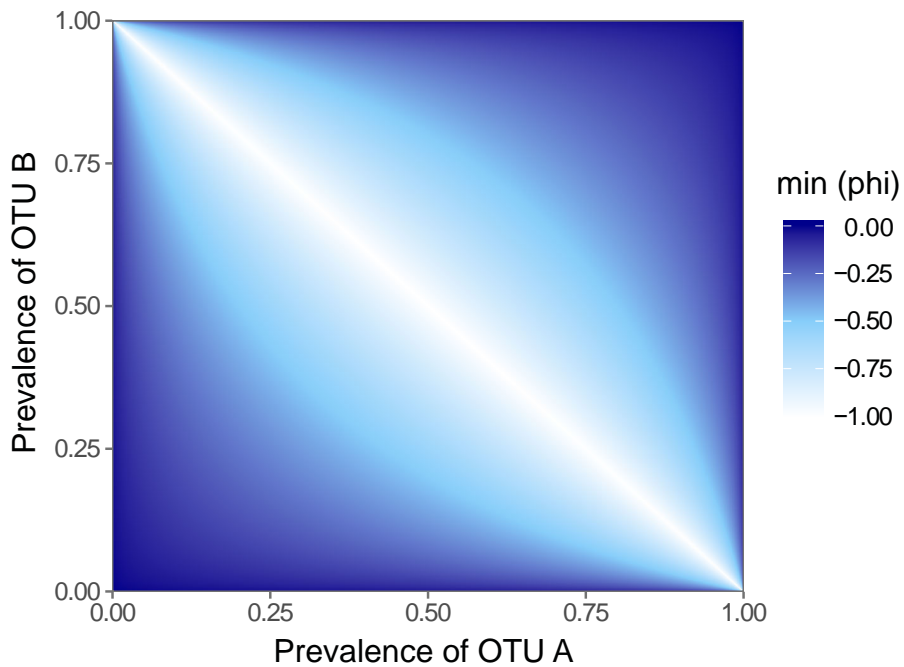**B**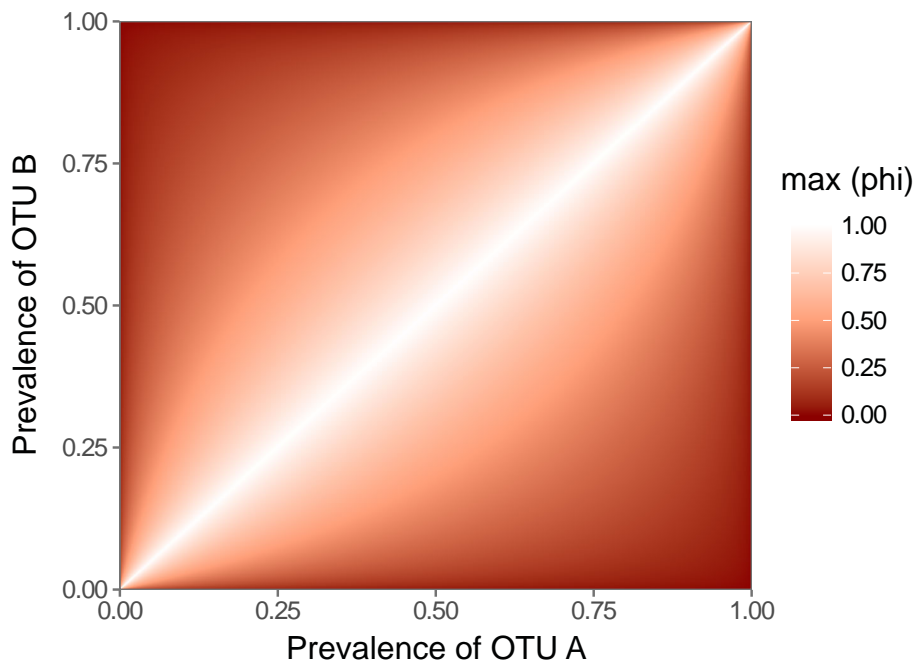

Supplement: S1 Fig — Minimum (A) and maximum (B) of the Phi coefficient as a function of prevalence. Computed from Eq (3). (PDF) [file pone.0200458.s001.pdf]

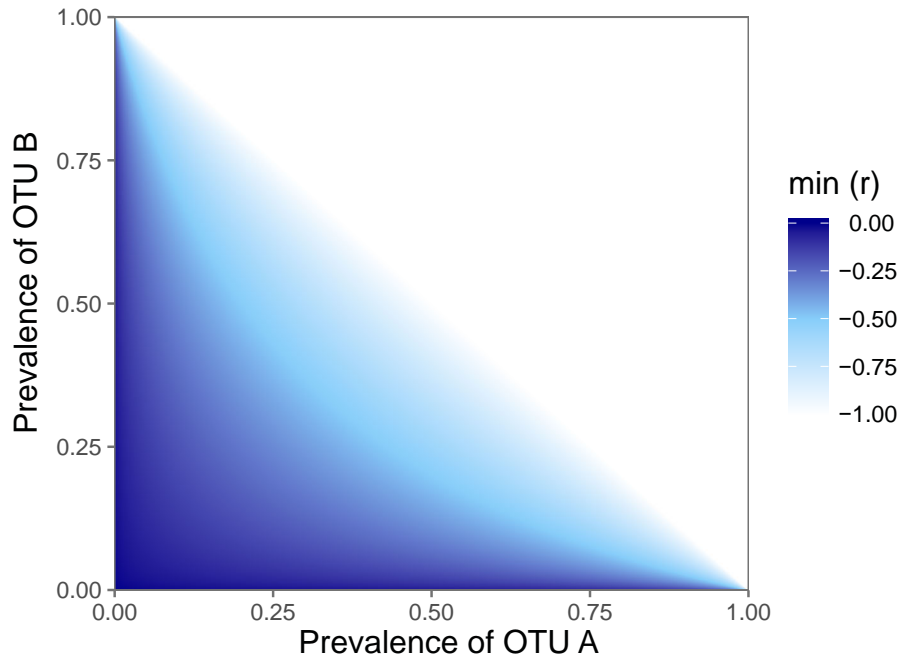

Supplement: S2 Fig — Minimum of the Pearson correlation coefficient r as a function of prevalence. Computed from Eq (6). (PDF) [file pone.0200458.s002.pdf]

**A**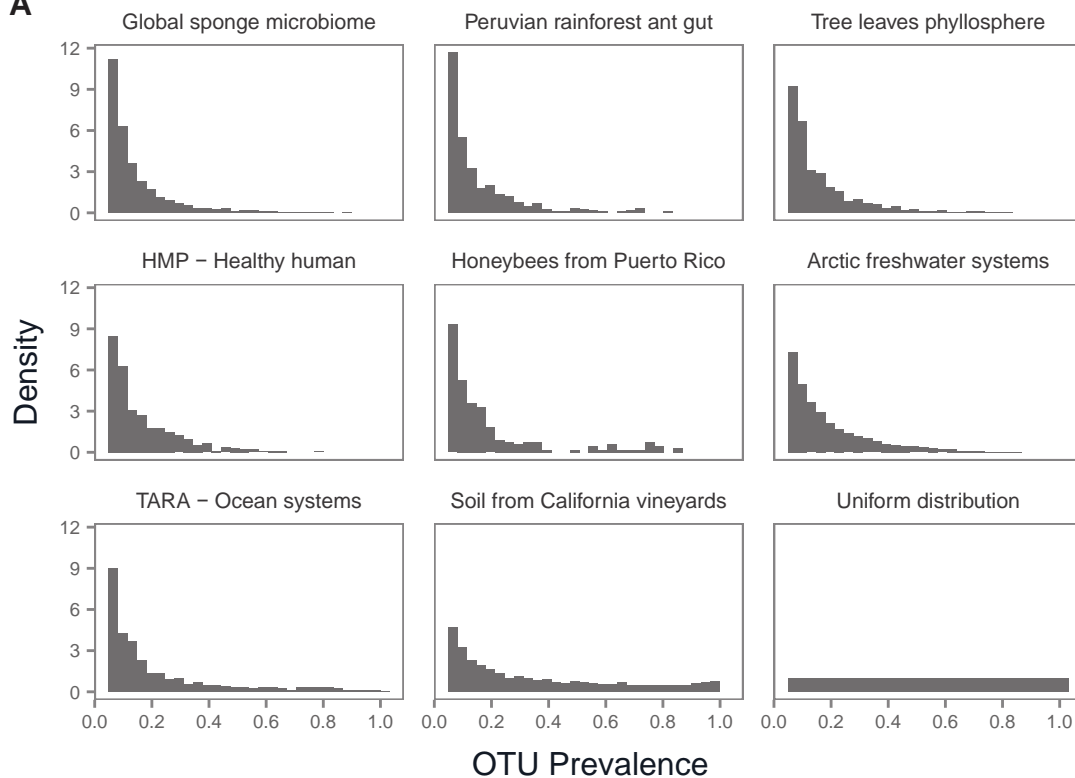**B**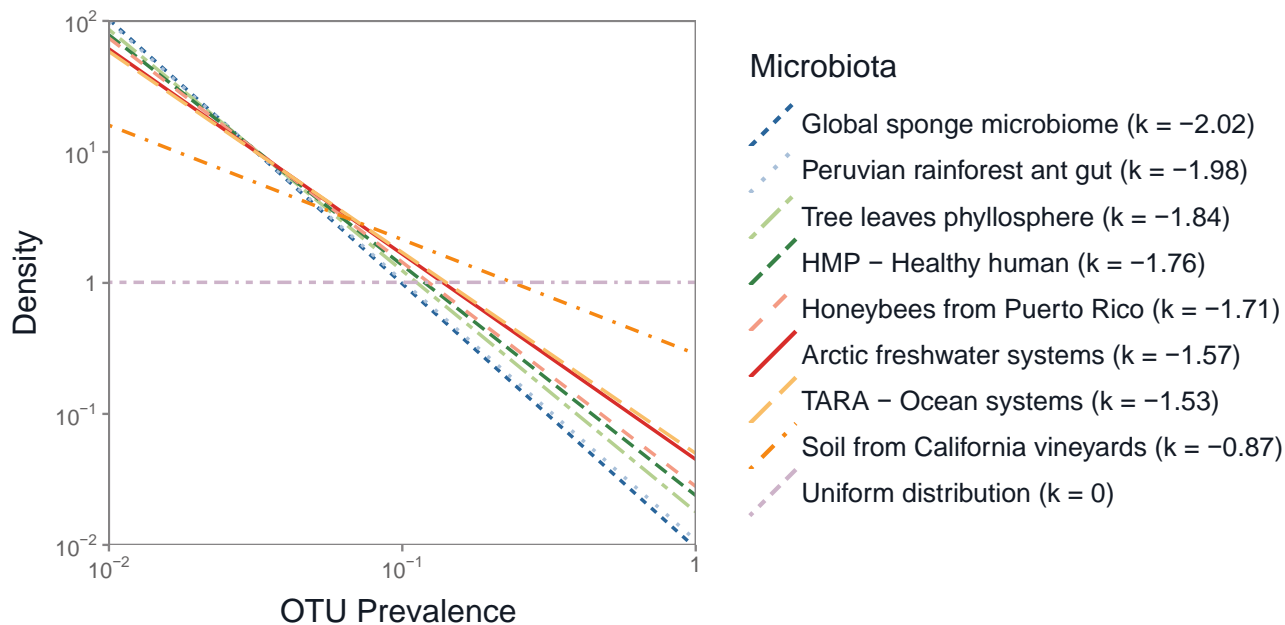

Supplement: S3 Fig — (A) Histograms of OTU prevalence in several microbiota characterized by 16S rRNA sequencing. The microbiota are described in Part E in S1 Appendix. (B) Probability density function of the same prevalence data (log-log scale), which were fitted to a truncated power law distribution; the power law coefficient k was estimated by maximizing log-likelihood. (PDF) [file pone.0200458.s003.pdf]

**A**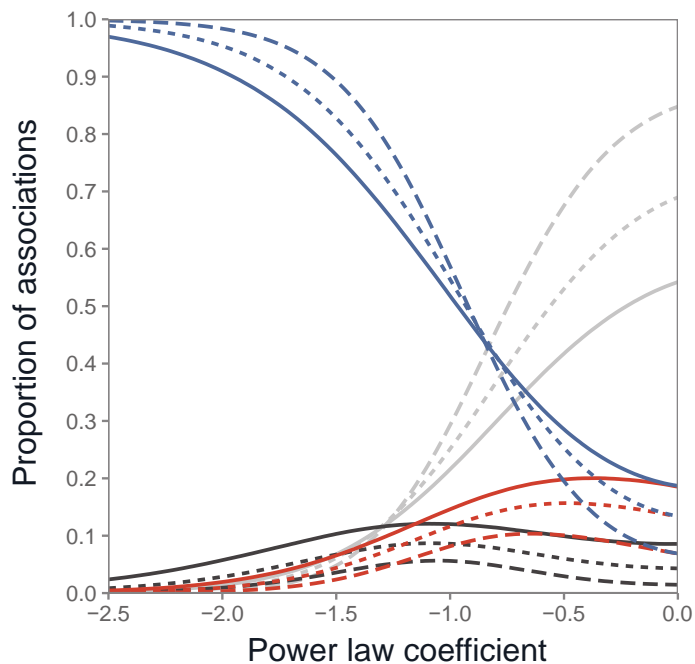**B**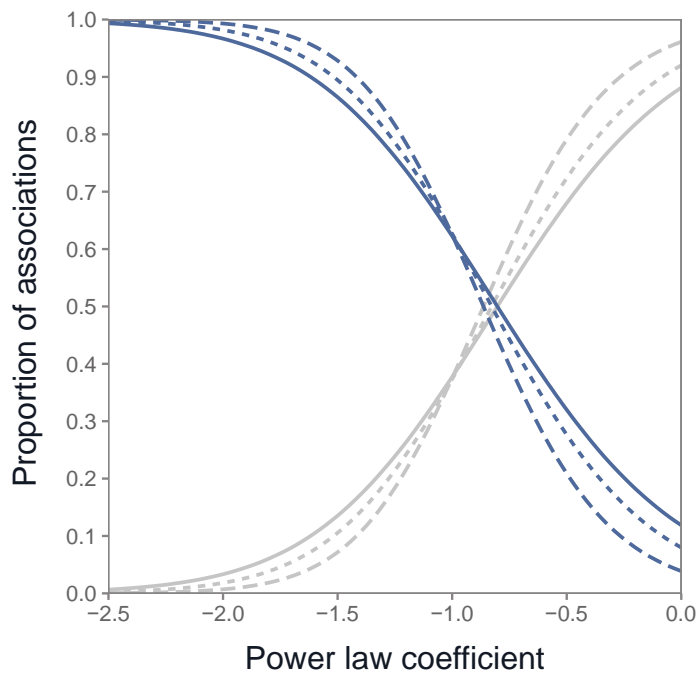

Supplement: S4 Fig — Proportion of testable associations as a function of k when N = 50, 100, or 300 for the occurrence data (A) and for the read abundance data (B). (PDF) [file pone.0200458.s004.pdf]
