# Supplementary material for: Rarity of microbial species: In search of reliable associations
Source: PLoS One. 2019 Mar 15;14(3):e0200458. doi: 10.1371/journal.pone.0200458 (PMC6420159; doi:10.1371/journal.pone.0200458)
Supplement: S1 Appendix — 1. Notation and decomposition of variance and covariance. 2. Threshold method for binary data. 3. Threshold method for quantitative data. 4. Similarity of the Phi and Pearson correlation coefficients. 5. Distribution of OTU prevalence in real microbiota. (PDF) [file pone.0200458.s005.pdf]

Rarity of microbial species: In search of reliable associations

Arnaud Cougoul, Xavier Bailly, Gwenaël Vourc'h, Patrick Gasqui

S1 Appendix. Supplementary Material

Contents

A. Notation and decomposition of variance and covariance 2
B. Threshold method for binary data 3
1. Measure of associations for binary data 3
2. Bounds of the Phi coefficient as a function of prevalence 4
3. Distribution of the Phi coefficient under the null hypothesis of independence 4
4. Determining the testability of occurrence-based associations 5
5. Proportion of associations in each testability zone 7
6. Defining testability zones using a Monte Carlo method 8
7. Testability limits on Fisher's exact test 8
C. Threshold method for quantitative data 9
1. Introduction 10
2. Determining the lower bound of the Pearson correlation coefficient 11
3. Maximising the inverse coefficient of variation 11
4. Determining the minimum Pearson correlation coefficient when there are many zeros 13
5. Constraints on the testability of the Pearson correlation coefficient 13
6. Proportion of associations in each testability zone 14
7. Spearman correlation invariance 14
8. Data transformation 15
D. Similarity of the Phi and Pearson correlation coefficients 16
1. Testability constraints on occurrence and abundance data 16
2. Correlation between Phi and Pearson coefficients 16
E. Distribution of OTU prevalence in real microbiota 17
References 17

Supplementary Material

In the supplementary material below, we describe how we established our thresholds for occurrence data (i.e., represented by binary variables) and read abundance data (i.e., represented by positive continuous variables). We then discuss the link between the two threshold types. Finally, we describe the 16S data from several microbial communities that we used to characterise prevalence patterns.
First, we present the notation and decomposition of variance and covariance as a function of OTU co-occurrence.

A. Notation and decomposition of variance and covariance

We consider two OTUs whose abundances are modelled by two random variables, X_A and X_B (Tables 1 a and b). Our threshold is based on presence or absence of OTU, so we created a contingency table whose categories are defined by variable presence or absence.

	$X_B = 0$	$X_B \neq 0$	Total
$X_A = 0$	N_{00}	N_{01}	$\overline{N_A} = N - N_A$
$X_A \neq 0$	N_{10}	N_{11}	N_A
Total	$\overline{N_B} = N - N_B$	N_B	N

Table 1a. Contingency table of the presence/absence of two OTU read-abundance variables X_A and X_B where the entries are sample counts.

	$X_B = 0$	$X_B \neq 0$	Total
$X_A = 0$	P_{00}	P_{01}	$\overline{P_A} = 1 - P_A$
$X_A \neq 0$	P_{10}	P_{11}	P_A
Total	$\overline{P_B} = 1 - P_B$	P_B	1

Table 1b. Contingency table of the presence/absence of two OTU read-abundance variables X_A and X_B where the entries are proportions.

N is the number of microbiota samples; N_{00} is the number of co-absences of X_A and X_B; N_{11} is the number of co-occurrences of X_A and X_B; and $P_{11} = N_{11}/N$ is the proportion of co-occurrences of the two OTUs. $P_A = N_A/N$ and $P_B = N_B/N$ are the marginal probabilities of X_A and X_B, respectively (i.e., individual OTU prevalence). Since the OTUs are observed at least once, $P_A, P_B \in [1/N, 1]$.

We can calculate the mean and estimated variance of X_A and X_B using the non-zero values of X_A or X_B. Consequently, $\mu_{X_A} = P_A \; \mu_{X_A | X_A \neq 0}$, and $\mu_{X_B} = P_B \; \mu_{X_B | X_B \neq 0}$.

The estimated variances of X_A and X_B can be calculated as follows:

$$\begin{aligned} {\sigma_{X_A}}^2 = \widehat{Var}(X_A) = \frac{1}{N} \sum\nolimits_N \left(X_A - \mu_{X_A}\right)^2 = P_A \; \left(\sigma_{X_A | X_A \neq 0}\right)^2 + \; P_A \; \overline{P_A} \; \left(\mu_{X_A | X_A \neq 0}\right)^2 \\ {\sigma_{X_B}}^2 = \widehat{Var}(X_B) = \frac{1}{N} \sum\nolimits_N \left(X_B - \mu_{X_B}\right)^2 = P_B \; \left(\sigma_{X_B | X_B \neq 0}\right)^2 + P_B \; \overline{P_B} \; \left(\mu_{X_B | X_B \neq 0}\right)^2 \end{aligned} \quad (1)$$

The estimated covariance of X_A and X_B can be decomposed based on whether or not X_A and X_B co-occur (i.e., X_A and X_B are non-null or not). If $\widehat{Cov}(X_A, X_B) = \mu_{X_A \times X_B} - \mu_{X_A} \times \mu_{X_B}$, then

$$\widehat{Cov}(X_A, X_B) = \overbrace{\left[P_{11}\ \widehat{Cov}\ (X_A, X_B)_{\mid X_A, X_B \neq 0}\right]}^{\text{"exclusively quantitative" covariance}} + \underbrace{\left[P_{11}\left(\mu_{X_A \mid X_A, X_B \neq 0} \times \mu_{X_B \mid X_A, X_B \neq 0}\right) - \left(\mu_{X_A} \times \mu_{X_B}\right)\right]}_{\text{"qualitative" covariance}} \quad (2)$$

"Exclusively quantitative" covariance

When the data are reduced into binary variables, $\widehat{Cov}\ (X_A, X_B)_{\mid X_A, X_B \neq 0} = 0$ because $\{X_A \mid X_A, X_B \neq 0\}$ and $\{X_B \mid X_A, X_B \neq 0\}$ are constants. Then $\left[P_{11}\ \widehat{Cov}\ (X_A, X_B)_{\mid X_A, X_B \neq 0}\right]$ is part of the covariance of X_A and X_B only because of the quantitative aspect of data.

"Qualitative" covariance

The second part of the covariance $\left[P_{11}\left(\mu_{X_A \mid X_A, X_B \neq 0} \times \mu_{X_B \mid X_A, X_B \neq 0}\right) - \left(\mu_{X_A} \times \mu_{X_B}\right)\right]$ is the difference between the mean product for the whole population and the mean product for the co-occurring elements only. Consequently, it can be explained by OTU co-occurrences (qualitative in nature).

When the data are reduced into binary variables (based on equations (1) and (2)):

$$\widehat{Cov}(X_A, X_B) = P_{11}\left(\mu_{X_A \mid X_A, X_B \neq 0} \times \mu_{X_B \mid X_A, X_B \neq 0}\right) - \left(\mu_{X_A} \times \mu_{X_B}\right) = P_{11} - P_A\ P_B$$

$${\sigma_{X_A}}^2 = P_A\ {\sigma_{X_A \neq 0}}^2 + P_A\ (1 - P_A)\ {\mu_{X_A \neq 0}}^2 = P_A\ \overline{P_A} \text{ and } {\sigma_{X_B}}^2 = P_B\ \overline{P_B}.$$

Therefore, the correlation of X_A and X_B, $cor(X_A, X_B) = \frac{\widehat{Cov}(X_A, X_B)}{\sigma_{X_A}\ \sigma_{X_B}}$, will depend only on P_{11}, P_A, and P_B.

B. Threshold method for binary data

Our method is based on the properties of discrete statistics. As binary data are discrete data, statistical tests have discrete distributions, as do *p*-values. Moreover, the minimum observable *p*-value for fixed marginal values can be higher than the alpha level (usually set to 5%), which means the test yields useless results [1,2]. In other words, for two OTUs with fixed prevalence, if all the possible values of an association index fall within the expected confidence interval, the association is simply not testable. Below, we will illustrate how OTU prevalence can thus shape potential correlations.

In this section, we detail how we developed our threshold method for binary data (i.e., OTU occurrence). First, we describe the association index used and show that it is bounded. Second, we present how we defined its testability. Third, we examine the consequences of our threshold method for network inference. Fourth, we present the testability limits on Fisher's exact test as a function of prevalence.

1. Measure of associations for binary data

The combinatorics that ensue from the hypergeometric law provide only simulated solutions for determining the testability of associations. In contrast, the Phi coefficient [3] can be used to establish equations for exploring association testability and give an analytical solution. The Phi coefficient is mathematically related to the common chi-square test. Since Fisher's exact test and Pearson's chi-square

test are asymptotically equivalent, we used the Phi coefficient as the basis for our threshold method. Moreover, we showed that the testability results were equivalent for both tests (see section A.7 and S1 Fig 3). Phi is also equivalent to the Pearson correlation coefficient in situations with binary data (coded by 0 and 1), a property that was helpful when extending our threshold method to quantitative situations (see sections 3 and 4).

Consider two random binary variables, $\widetilde{X_A}$ and $\widetilde{X_B}$, which represent the presence or absence of two OTUs. Working from Table 1, the Phi coefficient for the association between $\widetilde{X_A}$ and $\widetilde{X_B}$ is calculated as follows:

$$\phi = \frac{P_{11} - P_A P_B}{\sqrt{P_A \overline{P_A} P_B \overline{P_B}}} \text{ if } P_A,\ P_B \in \left]0,1\right[\text{, and } \phi = 0 \text{ if not} \quad (3)$$

2. Bounds of the Phi coefficient as a function of prevalence

Based on the Boole–Fréchet inequality for logical conjunction, for the marginal probabilities $P_A,\ P_B \in \left]0,1\right[$, it follows that

$$max(0,\ P_A + P_B - 1) \ \leq\ P_{11} \ \leq\ min(P_A,\ P_B) \quad (4)$$

Given equations (3) and (4) and because ϕ is a continuous and monotonic function of P_{11}:

$$-1 \ \leq\ \phi_{min} \ \leq\ \phi \ \leq\ \phi_{max} \ \leq\ +1 \quad (5)$$

where

$$\phi_{min} = max\left(-\left(\frac{P_A\, P_B}{\overline{P_A}\ \overline{P_B}}\right)^{1/2},\ -\left(\frac{\overline{P_A}\ \overline{P_B}}{P_A\, P_B}\right)^{1/2}\right) \quad (5a)$$

$$\phi_{max} = min\left(\left(\frac{P_A\,\overline{P_B}}{P_B\,\overline{P_A}}\right)^{1/2},\ \left(\frac{P_B\,\overline{P_A}}{P_A\,\overline{P_B}}\right)^{1/2}\right) \quad (5b)$$

[4]

Therefore, ϕ is bounded and ϕ_{min} and ϕ_{max} depend exclusively on P_A and P_B.

3. Distribution of the Phi coefficient under the null hypothesis of independence

Under the null hypothesis (H_0) that the occurrences of two OTUs, $\widetilde{X_A}$ and $\widetilde{X_B}$, are independent, ϕ can be determined thanks to the Pearson's chi-squared test: $\phi^2 \ =\ \chi^2/N$, where N is the total number of observations and χ^2 is the chi-squared statistic for a 2x2 contingency table whose data follow a chi-squared distribution and for which there is 1 degree of freedom [5].

Since we know the distribution of ϕ, we can obtain the confidence interval at an alpha level of α. The confidence interval of a χ_1^2 distribution is $CI_{1-\alpha}\,(\chi_1^2) = [0, b]$, where b is defined by $P(\chi_1^2 > b) = \alpha$ (e.g., for $\alpha = 5\%$, $b \approx 1.96^2 \approx 3.84$).

The confidence interval of ϕ at an alpha level of α can be calculated as follows:

$$CI_{1-\alpha}\,(\phi) = \left[-\sqrt{K}, \sqrt{K}\,\right] \text{, where } K = {}^{b}/_{N} \quad (6)$$

4. Determining the testability of occurrence-based associations

We now examine the testability of the Phi coefficients calculated from pairs of OTU prevalence values. We do so by determining if the extrema of Phi occur within the confidence interval. There are two ways in which we may have trouble detecting significant associations:

A) If $\phi_{min} > -\sqrt{K}$, then we will not be able to detect a significant negative association.
B) If $\phi_{max} < \sqrt{K}$, then we will not be able to detect a significant positive association.

As ϕ_{min} and ϕ_{max} depend exclusively on P_A and P_B, we now consider the conditions under which P_A and P_B adopt problematic values.

We can split the first case (**A**) in two subcases because ϕ_{min} can have two different values depending on the specific values of P_A and P_B:

A1) If $P_A + P_B < 1$, then $max(0,\ P_A + P_B - 1) = 0$. Based on equations (3), (4), and (5a),

$$\phi_{min} = -\left(\frac{P_A\,P_B}{\overline{P_A}\ \overline{P_B}}\right)^{1/2}$$

A2) If $P_A + P_B \geq 1$, $max(0,\ P_A + P_B - 1) = P_A + P_B - 1$. Based on equations (3), (4), (5a),

$$\phi_{min} = -\left(\frac{\overline{P_A}\ \overline{P_B}}{P_A\,P_B}\right)^{1/2}$$

We can then resolve the inequation $\phi_{min} > -\sqrt{K}$.

A1) For $P_A + P_B < 1,\ \phi_{min} > -\sqrt{K} \quad \Leftrightarrow \quad \left(\frac{P_A\,P_B}{\overline{P_A}\ \overline{P_B}}\right)^{\frac{1}{2}} < \sqrt{K}$

$\Leftrightarrow \quad \frac{P_A\,P_B}{(1-P_A)(1-P_B)} < K$ (all variables are positive)

$$\Leftrightarrow \quad P_B < \frac{1-P_A}{1+\frac{1-K}{K}P_A} \tag{7}$$

A2) For $P_A + P_B \geq 1,\ \phi_{min} > -\sqrt{K} \quad \Leftrightarrow \quad \frac{(1-P_A)(1-P_B)}{P_A\,P_B} < K$

$$\Leftrightarrow \quad P_B > \frac{-1+P_A}{-1+(1-K)P_A} \tag{8}$$

If inequations (7) or (8) are true, a negative association cannot be detected.

The second case (**B**) can be similarly split up because ϕ_{max} can also have two values:

B1) If $P_A \leq P_B$, then $min(P_A,\ P_B) = P_A$. Based on equations (3), (4), and (5b),

$$\phi_{max} = \left(\frac{P_A\,\overline{P_B}}{P_B\,\overline{P_A}}\right)^{1/2}$$

B2) If $P_A \geq P_B$, then $min(P_A,\ P_B) = P_B$. Based on equations (3), (4), and (5b),

$$\phi_{max} = \left(\frac{P_B\,\overline{P_A}}{P_A\,\overline{P_B}}\right)^{1/2}$$

We can now solve the inequation $\phi_{max} < \sqrt{K}$.

B1) If $P_A \leq P_B$, $\phi_{max} < \sqrt{K} \Leftrightarrow \frac{P_A(1-P_B)}{P_B(1-P_A)} < K$

$$\Leftrightarrow P_B > \frac{P_A}{K+(1-K)P_A} \tag{9}$$

B2) If $P_A \geq P_B$, $\phi_{max} < \sqrt{K} \Leftrightarrow \frac{P_B(1-P_A)}{P_A(1-P_B)} < K$

$$\Leftrightarrow P_B < \frac{P_A}{\frac{1}{K}+\frac{K-1}{K}P_A} \tag{10}$$

If inequations (9) or (10) are true, a positive association cannot be detected.

Using the four inequations (7), (8), (9), and (10), we can delimit zones within which there is full, partial, or no testability. The characteristics of the tests in these zones will be detailed in the introduction to the next section.

For the two OTUs, P_A and P_B form a $[1/N, 1]^2$ square (Figure 1 below); $1/N$ is the smallest observable value. The testability zones in this square can be defined using four border functions that result from the inequations:

$$F_1(x) = \frac{1-x}{1+\frac{1-K}{K}x};\ \ F_2(x) = \frac{-1+x}{-1+(1-K)x}; F_3(x) = \frac{x}{K+(1-K)x};\ \ F_4(x) = \frac{x}{\frac{1}{K}+\frac{K-1}{K}x} \tag{11}$$

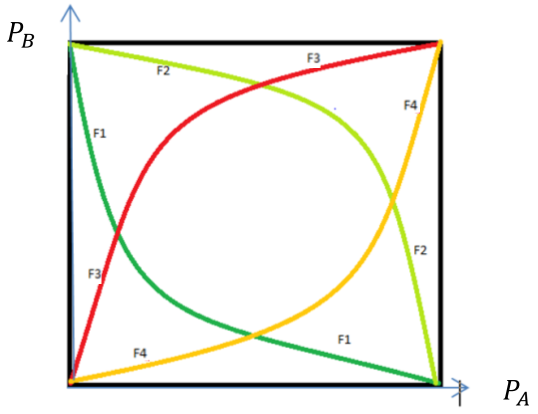

Figure 1. The four border functions delimiting testability

Emerging from these border functions are four graph intersections that are defined by:

$$\begin{aligned} F_1(x) = F_3(x) &= \frac{1}{2} \text{ at } x = \frac{K}{K+1} \\ F_2(x) = F_4(x) &= \frac{1}{2} \text{ at } x = \frac{1}{K+1} \\ F_2(x) = F_3(x) &= \frac{K}{K+1} \text{ at } x = \frac{1}{2} \\ F_1(x) = F_4(x) &= \frac{1}{K+1} \text{ at } x = \frac{1}{2} \end{aligned} \tag{12}$$

5. Proportion of associations in each testability zone

The zones defined by the border functions (11) contain different proportions of associations that can be categorised as fully testable, partially testable, or non-testable using our threshold method. The first zone, $A_{bilateral}$, contains associations for which both positive and negative correlations can be reliably tested. The second zone, $A_{unilateral}$, contains associations for which only positive correlations can be reliably tested (subzone $A_{positive}$) and for which only negative correlations can be reliably tested (subzone $A_{negative}$). Finally, the third zone, $A_{irrelevant}$, contains associations that cannot be reliably tested at all.

The distribution of prevalence values is treated as identical for all OTUs. Therefore, P_A and P_B have the same distribution and play symmetrical roles. However, these distribution patterns are not necessarily uniform. We examined two types of distributions—the uniform distribution and the truncated power law distribution; the latter fit the prevalence patterns of OTUs in real microbiota (see section 5).

For the uniform distribution of prevalence, the probability density function is

$$f(x) = \begin{cases} \dfrac{1}{1-\dfrac{1}{N}} = \dfrac{N}{N-1} & if\ \dfrac{1}{N} \leq x \leq 1 \\ 0 & if\ not \end{cases} \tag{13}$$

For the truncated power law distribution of prevalence, the probability density function is

$$f(x) = \begin{cases} Cx^k & if\ \dfrac{1}{N} \leq x \leq 1 \\ 0 & if\ not \end{cases} \tag{14}$$

and, following normalization, we arrive at $\int_{\frac{1}{N}}^{1} f(x)\,dx = C\frac{\left[x^{k+1}\right]_{1/N}^{1}}{k+1} = 1$, so $C = \frac{k+1}{1-\left(\frac{1}{N}\right)^{k+1}}$.

When $k = 0$, we have a uniform distribution with the interval $\left[\frac{1}{N}, 1\right]$.

To computationally define the different zones, analytical formulas can be used in the case of the uniform distribution but not in the case of the power law distribution. Consequently, in the latter situation, we chose to proceed by numerical integration. Since the current form of the R function *integrate* (in the stats package) does not deal well with the power law, we used a Monte Carlo approach. This consisted of generating random prevalence values in accordance with the observed prevalence distribution (see section 2.6) and counting how many fell within each of the zones.

To simplify the zone-defining equations below, we have used the following notation:

$(F_1 +)$: "$y > F_1(x)$" and $(F_1 -)$: "$y < F_1(x)$"

and the same notation applies in the cases of F_2, F_3 and F_4.

$\wedge$ denotes the logical conjunctions.

From the four inequations (7, 8, 9, 10) and the border function (11), the proportions of associations that fall within each zone are determined as follows:

$$A_{bilateral} = \iint_{\{(F_1+)\,\wedge\,(F_4+)\,\wedge\,(F_2-)\,\wedge\,(F_3-)\}} f(x)f(y)\,\mathrm{d}x\mathrm{d}y$$

$$A_{positive} = \iint_{\{(F_3-)\,\wedge\,(F_1-)\,\wedge\,(F_4+)\}} f(x)f(y)\,\mathrm{d}x\mathrm{d}y + \iint_{\{(F_3-)\,\wedge\,(F_2+)\,\wedge\,(F_4+)\}} f(x)f(y)\,\mathrm{d}x\mathrm{d}y$$

$$A_{negative} = \iint_{\{(F_1+)\,\wedge\,(F_3+)\,\wedge\,(F_2-)\}} f(x)f(y)\,\mathrm{d}x\mathrm{d}y + \iint_{\{(F_1+)\,\wedge\,(F_4-)\,\wedge\,(F_2-)\}} f(x)f(y)\,\mathrm{d}x\mathrm{d}y$$

$$A_{irrelevant} = \iint_{\{(F_3+)\,\wedge\,(F_1-)\}} f(x)f(y)\,\mathrm{d}x\mathrm{d}y + \iint_{\{(F_2+)\,\wedge\,(F_4-)\}} f(x)f(y)\,\mathrm{d}x\mathrm{d}y + \iint_{\{(F_2+)\,\wedge\,(F_3+)\}} f(x)f(y)\,\mathrm{d}x\mathrm{d}y + \iint_{\{(F_1-)\,\wedge\,(F_4-)\}} f(x)f(y)\,\mathrm{d}x\mathrm{d}y$$

$$A_{unilateral} = A_{positive} + A_{negative}$$

$$A_{bilateral} + A_{unilateral} + A_{irrelevant} = \iint f(x)f(y)\,\mathrm{d}x\mathrm{d}y = 1$$

6. Defining testability zones using a Monte Carlo method

To compute Monte Carlo integrations, it is necessary to generate random prevalence values using the observed distribution of prevalence. For the uniform distribution, many pseudorandom number generators exist. However, for the truncated power law distribution, we had to employ an inverse transformation method that is rooted in the following property:

If V follows a power law, then $F(V) = U$ is uniformly distributed (interval of [0,1]) and $F^{-1}(U) = V$.

We therefore needed to define the inverse cumulative distribution function. Let F be the cumulative distribution function of the truncated power law distribution as defined in (14).

If $F(x) = \int_{\frac{1}{N}}^{x} f(t)\,dt = C\int_{\frac{1}{N}}^{x} t^k\,dt = \frac{C}{k+1}\left(x^{k+1} - \left(\frac{1}{N}\right)^{k+1}\right) = \frac{x^{k+1}-\left(\frac{1}{N}\right)^{k+1}}{1-\left(\frac{1}{N}\right)^{k+1}}$,

then $F^{-1}(x) = \left(\left(1 - \left(\frac{1}{N}\right)^{k+1}\right)x + \left(\frac{1}{N}\right)^{k+1}\right)^{\frac{1}{k+1}}$.

We can then generate a power law distribution from a uniform distribution using the following equation:

$$V = \left(\left(1 - \left(\frac{1}{N}\right)^{k+1}\right)U + \left(\frac{1}{N}\right)^{k+1}\right)^{\frac{1}{k+1}} \tag{15}$$

7. Testability limits on Fisher's exact test

Co-occurrence networks are commonly reconstructed using the hypergeometric law that underlies Fisher's exact test [6–8].

From an observed 2x2 contingency table (Table 1), Fisher showed that the probability P of obtaining such a set was given by the hypergeometric distribution:

$$P = \frac{\binom{N_{00}+N_{01}}{N_{00}} + \binom{N_{10}+N_{11}}{N_{10}}}{\binom{N}{N_{00}+N_{10}}} = \frac{(N_{00}+N_{01})!\ (N_{10}+N_{11})!\ (N_{00}+N_{10})!\ (N_{01}+N_{11})!}{N_{00}!\ N_{01}!\ N_{10}!\ N_{11}!\,N!} \quad (16)$$

where $\binom{n}{k}$ is the binomial coefficient and ! indicates the factorial.

This equation can be written according to N_A, N_B, N and N_{11}:

$$P = \frac{\binom{N-N_A}{N_{11}+(N-N_A)+(N-N_B)-N} + \binom{N_A}{N-N_{11}-(N-N_A)}}{\binom{N}{N-N_B}} = \frac{\binom{N-N_A}{N_B-N_{11}} + \binom{N_A}{N_{11}}}{\binom{N}{N_B}} \quad (17)$$

Based on the Boole–Fréchet inequality for logical conjunction, for the marginal counts $N_A,\ N_B \in\ ]0, N[$, it follows that

$$max(0,\ N_A + N_B - \mathrm{N})\ \leq\ N_{11}\ \leq\ min(N_A,\ N_B) \quad (18)$$

We have two extreme situations:

a) Observe the minimum number of co-occurrences, $N_{11} = min(N_{11}) = max(0,\ N_A + N_B - \mathrm{N})$

b) Observe the maximum number of co-occurrences, $N_{11} = max(N_{11}) = min(N_A,\ N_B)$

We can calculate the probability P associated with these two situations **a)** and **b)**. A bilateral test can also be performed. As in the fisher.test function of R, the p-value is computed by summing the probability for all table with probabilities less than or equal to that of the observed table.

For two given OTUs with prevalence $P_A = \frac{N_A}{N}$ and $P_B = \frac{N_B}{N}$, we have 4 possibilities in the testability limits on Fisher's exact test:

- If the p-values associated with the two configurations **a)** and **b)** are lower than the alpha level (5%), the two extremes situations **a)** and **b)** correspond to significant associations. We have no limit on the test.
- If the p-value associated with the configuration **a)** is greater than the alpha level, then we will not be able to detect a significant negative association.
- If the p-value associated with the configuration **b)** is greater than the alpha level, then we will not be able to detect a significant positive association.
- If the p-values associated with the configurations **a)** and **b)** are greater than the alpha level, then we will not be able to detect a significant positive or negative association.

C. Threshold method for quantitative data

In this section, we detail how we developed our threshold method for quantitative data (i.e., OTU read abundance). First, we introduce our system of notation and the primary elements of our proof. Second, we present the situation, in which correlations are bounded by an excess of zeroes, and describe the minimum correlation value. Third, we show how we defined association testability. Finally, we examine the consequences of our threshold method for network inference.

1. Introduction

In this section, X_A and X_B are two random variables that represent quantitative data. The Pearson correlation coefficient [9] is used to characterise the pairwise associations in OTU read abundance. We were specifically interested in understanding how the number of zeroes in the data could influence the correlation coefficient.

We use same notations as in section 1. $\overline{N_A}$ and $\overline{N_B}$ represent the number of zeros associated with X_A and X_B, respectively. N_{00} is the number of co-absences of X_A and X_B, and N_{11} is the number of co-occurrences.

Based on Table 1 and the Boole–Fréchet inequalities, we can deduce the following:

$$max(0;\ \overline{N_A} + \overline{N_B} - N) \leq N_{00} \leq min(\overline{N_A}; \overline{N_B}) \tag{19}$$

$$N_{11} = N - \overline{N_A} - \overline{N_B} + N_{00} \tag{20}$$

$$N_{11} \geq max(N - \overline{N_A} - \overline{N_B};\ 0) \tag{21}$$

For pairs of $\overline{N_A}$ and $\overline{N_B}$, we distinguish two cases:

i. $\boldsymbol{\overline{N_A} + \overline{N_B} \leq N}$

The number of zeros is sufficiently low such that there are no raw restrictions on possible correlations. Indeed, it is simple to build two non-restricted correlations that approach infimum -1 and supremum $+1$:

$$\begin{pmatrix} X_A \\ X_B \end{pmatrix} = \begin{pmatrix} \overbrace{0,\ 0,\ \dots,0,}^{\overline{N_A}} & \overbrace{a, a, \dots, a,}^{N_{11}} & 2a, 2a, \dots, 2a \\ 2a, 2a, \dots, 2a, & a, a, \dots, a, & \underbrace{0,\ 0,\ \dots,0}_{\overline{N_B}} \end{pmatrix} \text{ where } a > 0$$

In this case, the correlation coefficient is $r = -1$.

$$\begin{pmatrix} X_A \\ X_B \end{pmatrix} = \begin{pmatrix} \overbrace{0,0,\dots,0, \quad 0,0,\dots,0,}^{\overline{N_A}} & \overbrace{a, a, \dots, a}^{N_{11}} \\ \underbrace{0,0,\dots,0,}_{\overline{N_B}} \quad h, h, \dots, h, & a, a, \dots, a \end{pmatrix} \text{ where } a, h > 0$$

The correlation tends toward the supremum, $r \xrightarrow[h \to 0]{} +1$ or $r \xrightarrow[a \to +\infty]{} +1$.

ii. $\boldsymbol{\overline{N_A} + \overline{N_B} > N}$

Based on equations (19) and (21), $N_{00} \geq \overline{N_A} + \overline{N_B} - N > 0$ and $N_{11} \geq 0$. Consequently, N_{11} can equal zero, meaning that there are enough zeros associated with X_A and X_B that X_A and X_B may not co-occur. In this situation, information on quantitative correlations is degraded. We can prove that r, the Pearson correlation coefficient, has a minimum, r_{min}, that is different from -1:

$r_{min} \leq r \leq 1$,

where $r_{min} = -\left(\frac{N_A\, N_B}{\overline{N_A}\, \overline{N_B}}\right)^{1/2} > -1$

2. Determining the lower bound of the Pearson correlation coefficient

Given $\overline{N_A}$ and $\overline{N_B}$, we wished to determine the minimum possible correlation between X_A and X_B. We highlight that a lower bound of the Pearson correlation exists between two positive variables and prove that it can be reached under certain conditions.

For the association between X_A and X_B, the Pearson correlation coefficient is calculated as follows:

$$r = \frac{\widehat{Cov}(X_A, X_B)}{\sigma(X_A)\,\sigma(X_B)} = \frac{\mu(X_A X_B) - \mu(X_A)\,\mu(X_B)}{\sigma(X_A)\,\sigma(X_B)}$$

If $X_A, X_B \geq 0$, then $\mu(X_A X_B) \geq 0$ and

$$r \geq \frac{-\mu(X_A)\,\mu(X_B)}{\sigma(X_A)\,\sigma(X_B)} \tag{22}$$

where equality holds if and only if $\mu(X_1 X_2) = 0$

Consequently, the mean of $X_A X_B$ is null if and only if there are no co-occurrences. In other words,

$$\mu(X_A X_B) = 0 \text{ if and only if } N_{11} = 0 \tag{23}$$

- If $\mu(X_1 X_2) = 0$, then $\sum X_1 X_2 = 0$. Each element of the sum are positive then $\sum X_1 X_2 = 0$ imply that all elements are null and there are no co-occurrences (i.e., $N_{11} = 0$).
- If there are no co-occurrences, then $X_1 X_2 = 0$ and $\mu(X_1 X_2) = 0$.

From equations (22) and (23), we can conclude that

$$-\frac{\mu(X_A)\,\mu(X_B)}{\sigma(X_A)\,\sigma(X_B)} \leq r \tag{24}$$

where equality holds if and only if $N_{11} = 0$

Moreover, if $\overline{N_A} + \overline{N_B} \leq N$, then, from equation (21), we know that $N_{11} \neq 0$. Therefore,

$$N_{11} = 0 \Longrightarrow \overline{N_A} + \overline{N_B} > N \tag{25}$$

We now want to control $-\frac{\mu(X_A)\,\mu(X_B)}{\sigma(X_A)\,\sigma(X_B)}$ and find its minimum. We therefore maximise $\frac{\mu(X_A)}{\sigma(X_A)}$ and $\frac{\mu(X_B)}{\sigma(X_B)}$ separately. μ/σ corresponds to the inverse coefficient of variation.

3. Maximising the inverse coefficient of variation

Below, we illustrate how to maximise the inverse coefficient of variation for X_A. We will show that $\frac{\mu(X_A)}{\sigma(X_A)} \leq \sqrt{\frac{N_A}{\overline{N_A}}}$.

We can express variance using the König–Huygens formula:

$$\widehat{Var}(X_A) = \mu\left({X_A}^2\right) - \mu(X_A)^2$$

If $\mu(X_A) \neq 0$, then

$\frac{\widehat{Var}(X_A)}{\mu(X_A)^2} = \frac{\mu({X_A}^2)}{\mu(X_A)^2} - 1$ and $\frac{\widehat{Var}(X_A)}{\mu(X_A)^2} = \frac{\frac{1}{N}\sum_{i=1}^{N} {X_A}_i^2}{\left(\frac{1}{N}\sum_{i=1}^{N} {X_A}_i\right)^2} - 1$, which means

$$\frac{\widehat{Var}(X_A)}{\mu(X_A)^2} = N\frac{\sum_{i=1}^{N} {X_A}_i^2}{\left(\sum_{i=1}^{N} {X_A}_i\right)^2} - 1 \tag{26}$$

We are now interested in $\frac{\sum_{i=1}^{N} {X_A}_i^2}{\left(\sum_{i=1}^{N} {X_A}_i\right)^2}$, and we will show that $\frac{\sum_{i=1}^{N} {X_A}_i^2}{\left(\sum_{i=1}^{N} {X_A}_i\right)^2} \geq \frac{1}{N_A}$.

Let V, W be two vectors of $\mathbb{R}^N$. As per the Cauchy–Schwarz inequality,

$$\left(\sum_{i=1}^{N} V_i \times W_i\right)^2 \leq \sum_{i=1}^{N} V_i^2 \sum_{i=1}^{N} W_i^2$$

where equality holds if and only if V and W are collinear.

Let $V = Y$ be the vector of non-null elements of X_A (for Y, vector size is equal to N_A); $W = 1_{N_A}$, a constant vector of size N_A. In this case, the Cauchy–Schwarz inequality becomes the following:

$$\left(\sum_{i=1}^{N_A} Y_i \times 1\right)^2 \leq \sum_{i=1}^{N_A} Y_i^2 \sum_{i=1}^{N_A} 1^2$$

where equality holds if and only if $Y = \lambda \times 1_{N_A}$, where $\lambda > 0$ (i.e., Y is a constant vector).

As $\sum_{i=1}^{N_A} Y_i = \sum_{i=1}^{N} {X_A}_i$ and $\sum_{i=1}^{N_A} Y_i^2 = \sum_{i=1}^{N} {X_A}_i^2$,

$\left(\sum_{i=1}^{N} {X_A}_i\right)^2 \leq N_A \sum_{i=1}^{N} {X_A}_i^2$, then

$$\frac{\sum_{i=1}^{N} {X_A}_i^2}{\left(\sum_{i=1}^{N} {X_A}_i\right)^2} \geq \frac{1}{N_A} \tag{27}$$

where equality holds if and only if the non-null elements of X_A are constant.

Based on equations (26) and (27), we now observe that

$$\frac{\widehat{Var}(X_A)}{\mu(X_A)^2} \geq \frac{N}{N_A} - 1 \Leftrightarrow \frac{\widehat{Var}(X_A)}{\mu(X_A)^2} \geq \frac{N - N_A}{N_A} \Leftrightarrow \frac{\sqrt{\mu(X_A)^2}}{\sqrt{\widehat{Var}(X_A)}} \leq \frac{\sqrt{N_A}}{\sqrt{N - N_A}}$$

Finally,

$$\frac{\mu(X_A)}{\sigma(X_A)} \leq \frac{\sqrt{N_A}}{\sqrt{\overline{N_A}}} \tag{28}$$

where equality holds if and only if the non-null elements of X_A are constant.

The maximum occurs where $\frac{\mu(X_A)}{\sigma(X_A)}$ is $\sqrt{\frac{N_A}{\overline{N_A}}}$.

The approach is equivalent for X_B, so we can conclude that

$$\frac{\mu(X_B)}{\sigma(X_B)} \leq \frac{\sqrt{N_B}}{\sqrt{\overline{N_B}}} \tag{29}$$

where equality holds if and only if the non-null elements of X_B are constant.

4. Determining the minimum Pearson correlation coefficient when there are many zeros

Based on equations (24), (28), and (29),

$$-\sqrt{\frac{N_A N_B}{\overline{N_A}\,\overline{N_B}}} \leq r \tag{30}$$

where equality holds if and only if $N_{11} = 0$ and the non-null elements of X_A and X_B are constant.

It therefore stands to reason that

$$\text{if } \overline{N_A} + \overline{N_B} > N, \text{ then } -\sqrt{\frac{N_A N_B}{\overline{N_A}\,\overline{N_B}}} > -1 \tag{31}$$

$$\frac{N_A\,N_B}{\overline{N_A}\,\overline{N_B}} = \frac{(N-\overline{N_A})(N-\overline{N_B})}{\overline{N_A}\,\overline{N_B}} = \frac{N\left(N-(\overline{N_A}+\overline{N_B})\right)+\overline{N_A}\,\overline{N_B}}{\overline{N_A}\,\overline{N_B}} = \frac{N\left(N-(\overline{N_A}+\overline{N_B})\right)}{\overline{N_A}\,\overline{N_B}} + 1$$

If $\overline{N_A} + \overline{N_B} > N,\; N - (\overline{N_A} + \overline{N_B}) < 0$ and $\frac{N\left(N-(\overline{N_A}+\overline{N_B})\right)}{\overline{N_A}\,\overline{N_B}} + 1 < 1$

Therefore, $\frac{N_A\,N_B}{\overline{N_A}\,\overline{N_B}} < 1$ and $-\sqrt{\frac{N_A N_B}{\overline{N_A}\,\overline{N_B}}} > -1$.

Finally, based on equations (30) and (31), when $\overline{N_A} + \overline{N_B} > N$,

$$-1 < r_{min} \leq r \leq 1 \tag{32}$$

where r can attain $r_{min} = -\sqrt{\frac{N_A N_B}{\overline{N_A}\,\overline{N_B}}}$ if and only if $N_{11} = 0$ and the non-null elements of X_A and X_B are constant.

5. Constraints on the testability of the Pearson correlation coefficient

When X_A and X_B follow two uncorrelated normal distributions, $r \sim \frac{t}{\sqrt{N-2+t^2}}$, where t is a Student's t statistic with degrees of freedom $N-2$. We can then determine a confidence interval: $CI_{1-\alpha}(r) = \left[-\sqrt{K};\ \sqrt{K}\right]$, where K depends on α and N.

Returning to our measures of OTU prevalence, if $P_A = \frac{N_A}{N}$ and $P_B = \frac{N_B}{N}$, then $r_{min} = -\sqrt{\frac{N_A N_B}{\overline{N_A}\,\overline{N_B}}} = -\sqrt{\frac{P_A P_B}{\overline{P_A}\,\overline{P_B}}}$. The constraint is the same as in the case of binary data.

If r_{min} falls within the confidence interval, we can conclude that negative associations cannot be detected.

$$-\sqrt{\frac{P_A P_B}{\overline{P_A}\,\overline{P_B}}} > -\sqrt{K}$$
$$\Leftrightarrow \quad P_B < \frac{1 - P_A}{1 + \frac{1-K}{K} P_A} \tag{33}$$

Accordingly, if inequation (33) is true, then negative associations are not testable.

The border function that defines the testability zones in the square formed by $P_A \times P_B$ is as follows:

$$F_1(x) = \frac{1 - x}{1 + \frac{1-K}{K} x} \tag{34}$$

6. Proportion of associations in each testability zone

Using the border function (34), we observed that two zones existed. The first zone, $A_{bilateral}$, contains associations for which both positive and negative correlations can be reliably tested. The second zone, $A_{positive}$, contains associations for which only positive correlations can be reliably tested. As for the binary data (sections 2.5 and 2.6), we explored the testability of abundance-based associations using the uniform distribution and the truncated power law distribution. In the latter case, we again employed a Monte Carlo approach.

Based on the border function (34), the proportions of associations that fall within each zone can be determined as follows:

$$A_{bilateral} = \iint_{\{(F_1+)\}} f(x)f(y)\,\mathrm{d}x\mathrm{d}y$$

$$A_{positive} = \iint_{\{(F_1-)\}} f(x)f(y)\,\mathrm{d}x\mathrm{d}y$$

$$A_{bilateral} + A_{positive} = \iint f(x)f(y)\,\mathrm{d}x\mathrm{d}y = 1$$

(Same notation as in section 2.5)

7. Spearman correlation invariance

The Spearman correlation between two continuous variables X_A and X_B is calculated as follows:

$$\rho_{Spearman}(X_A, X_B) = r_{Pearson}(rg(X_A), rg(X_B))$$

where $rg(X)$ is the function that associates the ranks of X.

The identical values will be assigned to the average of their positions in the ascending order of the values, which is equivalent to averaging over all possible permutations.

If we call $\overline{N_A}$ the number of zeros in X_A, the $\overline{N_A}$ zero values will be identical values and will be assigned to the rank $mean(\{1, \dots, \overline{N_A}\})$, $\{1, \dots, \overline{N_A}\}$ being all possible rank values for these $\overline{N_A}$ null values.

As $mean(\{1, \dots, \overline{N_A}\} = \frac{\overline{N_A}\,(\overline{N_A}-1)}{2}$,

We are now interested by $Y_A = rg(X_A) - \frac{\overline{N_A}\,(\overline{N_A}-1)}{2}$ and $Y_B = rg(X_B) - \frac{\overline{N_B}\,(\overline{N_B}-1)}{2}$.

If $X_A = 0$, $rg(X_A) = \frac{\overline{N_A}\,(\overline{N_A}-1)}{2}$ and $Y_A = rg(X_A) - \frac{\overline{N_A}\,(\overline{N_A}-1)}{2} = 0$

Zeros of X_A are zeros of Y_A, and the same for X_B and Y_B.

Moreover,

$$r_{Pearson}(Y_A, Y_B) = r_{Pearson}\left(rg(X_A) - \frac{\overline{N_A}\,(\overline{N_A}-1)}{2},\ rg(X_B) - \frac{\overline{N_B}\,(\overline{N_B}-1)}{2}\right)$$

As correlation is invariant by translation:

$$r_{Pearson}(Y_A, Y_B) = r_{Pearson}(rg(X_A),\ rg(X_B)) = \rho_{Spearman}(X_A, X_B)$$

We thus constructed two variables Y_A and Y_B which:

- have the same null values than X_A and X_B.
- have a Pearson correlation equal to the Spearman correlation of X_A and X_B
- are two positive continuous variables with the same limitations on their Pearson correlation depending on prevalence as described in the part 3.5.

Thus, when we study Spearman correlation, we implicitly make a Pearson correlation with the same number of zeros and then the same limitations as we have previously mentioned.

8. Data transformation

Since the correlation is invariant by translation (see the paragraph above), if a positive transformation $t()$ transforms all the null values in a single value z_0, it suffices to study the correlation $cor(t(X_A) - z_0, t(X_B) - z_0)$ to return to the general problem. The limit on the testability of the correlation will be the same for this type of transformation.

For microbial data, this works for Total Sum Scaling (TSS) and rarefying.

The centered log ratio (clr), the cumulative sum scaling (CSS) and DESeq transformation use a pseudo-count that did not produce the theoretical results obtained, although the simulations show that the problem is still present for the clr transformations and this is also probably the case for the others.

Use of a pseudo count to avoid log(0) is not ideal because clustering results have been shown to be very sensitive to the choice of pseudo-count, due to the nonlinear nature of the log transform[10,11].

D. Similarity of the Phi and Pearson correlation coefficients

In this section, we show that testability constraints tend to be similar with both occurrence and abundance data. We also examine the degree of correlation between the correlation coefficients calculated using the two data types.

1. Testability constraints on occurrence and abundance data

The distribution of the correlation coefficient for two normally distributed independent variables is $r \sim \frac{t_{N-2}}{\sqrt{N-2+t_{N-2}^2}}$.

As $t_{N-2} \xrightarrow[N\to+\infty]{} \mathcal{N}(0,1)$ (i.e., there is distribution convergence) and $\frac{\sqrt{N-2+t_{N-2}^2}}{\sqrt{N}} \xrightarrow[N\to+\infty]{} 1$, then $r \xrightarrow[N\to+\infty]{} \frac{\mathcal{N}(0,1)}{\sqrt{N}}$. Since the distribution of the square of the Phi coefficient is $\phi^2 \sim \frac{\chi_1^2}{N} \sim \frac{\mathcal{N}(0,1)^2}{N}$ under the null hypothesis of independence, the Pearson correlation coefficient will asymptotically attain the same confidence interval as the Phi coefficient: their lower bounds converge upon $\sqrt{b/N}$ (sections 2.3 and 3.5).

We now underscore that the Phi and Pearson correlation coefficients have the same lower bound when the two OTUs have low levels of prevalence: $r_{min} = \phi_{min} = -\sqrt{\frac{P_A P_B}{\overline{P_A}\,\overline{P_B}}}$.

When N is large enough, the testability of positive associations will be the same for binary data and quantitative data. This pattern will be all the more pronounced given that, in real microbiota, OTU prevalence is greatly skewed to the right: positive associations represent the majority of associations to be tested.

2. Correlation between Phi and Pearson coefficients

In section 1, we showed that variance can be decomposed in a quantitative part and a qualitative part (equation (2)). Here, we use the results of a simulation to explore how the strength of the correlation between the values of the Phi coefficient and the Pearson coefficient is related to OTU prevalence. We are most interested in what happens when prevalence is low.

OTU abundances X_A and X_B are modelled by a zero-inflated Poisson (ZIP) distribution using the following probability mass function:

$$f(x) = \begin{cases} p_0 + (1-p_0).e^{-\lambda} & if\ x = 0 \\ (1-p_0).\frac{\lambda^x e^{-\lambda}}{x!} & if\ x = 1, 2 \ldots \end{cases}$$

where the probability of structural zeros, p_0, is the result of a Bernoulli process and λ is the mean of the Poisson portion of the distribution (i.e., the Poisson parameter). In the simulation, X_A and X_B had the same values for p_0 and λ.

The probability of structural zeros p_0 represents the complementary probability of prevalence P, i.e. $p_0 = 1 - P$. As p_0 increases (i.e., prevalence decreases), the correlation between the Phi coefficient and the Pearson coefficient increases (Figure 3A in the article). The correlation also strengthens as λ increases. When prevalence is below 0.25, the correlation is greater than 0.75 for all values of λ.

If OTU prevalence follows a ZIP distribution, we can conclude that the values of the Phi coefficient and the Pearson coefficient will be correlated, especially when OTU prevalence is low.

E. Distribution of OTU prevalence in real microbiota

To characterise actual OTU distribution patterns, we employed data from the QIITA database (qiita.ucsd.edu) and the TARA Ocean Project (ocean-microbiome.embl.de) [12]. The biom files were processed using the R package *biomformat*. We deliberately chose different kinds of microbiota so as to represent as wide a diversity of microbial communities as possible (Table 2). We used OTU rather than species tables.

The prevalence values were fitted to a truncated power law distribution as described by equation (14), and the power law coefficient k was estimated by maximizing the log-likelihood [13].

Source	Samples	OTUs	Median of Prevalence	Mean sequencing depth	Estimated k
Arctic freshwater systems (ID Qiita 1883)	3153	32347	0.004440216	47903.11	-1.567
Gut bacteria of Peruvian rainforest ants (ID Qiita 10343)	471	9819	0.004246285	34773.16	-1.981
HMP healthy human [14] (ID Qiita 1928)	6000	10730	0.0006666667	4538.797	-1.758
Honeybees from Puerto Rico (ID Qiita 1064)	387	3789	0.002583979	14974.18	-1.711
Soil from California vineyards (ID Qiita 10082)	237	13149	0.05907173	23479.96	-0.873
Sponge (ID Qiita 1740)	1403	24447	0.00427655	42056.75	-2.018
Tree leaves [15] (ID Qiita 396)	107	4218	0.01869159	936.7477	-1.841
TARA Ocean Project [12]	139	24798	0.02158273	34168.53	-1.534

Table 2. Sources of the microbiota we analysed and the associated number of samples, number of OTUs, and estimates of the power law coefficient k.

References

1. Tarone RE. A Modified Bonferroni Method for Discrete Data. Biometrics. 1990;46: 515. doi:10.2307/2531456

2. Carlson J, Heckerman D, Shani G. Estimating false discovery rates for contingency tables. Microsoft, Redmond, WA. 2009.

3. Yule GU. On the Methods of Measuring Association Between Two Attributes. J R Stat Soc. 1912;75: 579. doi:10.2307/2340126

4. Chaganty NR, Joe H. Range of correlation matrices for dependent Bernoulli random variables. Biometrika. 2006;93: 197–206. doi:10.1093/biomet/93.1.197

5. Guilford JP. The phi coefficient and chi square as indices of item validity. Psychometrika. 1941;6: 11–19. doi:10.1007/BF02288569

6. Chaffron S, Rehrauer H, Pernthaler J, von Mering C. A global network of coexisting microbes from environmental and whole-genome sequence data. Genome Res. 2010;20: 947–959. doi:10.1101/gr.104521.109

7. Faust K, Raes J. Microbial interactions: from networks to models. Nat Rev Microbiol. Nature Publishing Group; 2012;10: 538–550. doi:10.1038/nrmicro2832

8. Li C, Lim KMK, Chng KR, Nagarajan N. Predicting microbial interactions through computational approaches. Methods. Elsevier Inc.; 2016;102: 12–19. doi:10.1016/j.ymeth.2016.02.019

9. Pearson K. Mathematical Contributions to the Theory of Evolution. III. Regression, Heredity, and Panmixia. Philos Trans R Soc A Math Phys Eng Sci. 1896;187: 253–318. doi:10.1098/rsta.1896.0007

10. Costea PI, Zeller G, Sunagawa S, Bork P. A fair comparison. Nat Methods. Nature Publishing Group; 2014;11: 359–359. doi:10.1038/nmeth.2897

11. Paulson JN, Bravo HC, Pop M. Reply to: A fair comparison. Nat Methods. 2014;11: 359–360. doi:10.1038/nmeth.2898

12. Sunagawa S, Coelho LP, Chaffron S, Kultima JR, Labadie K, Salazar G, et al. Structure and function of the global ocean microbiome. Science (80-). 2015;348: 1261359–1261359. doi:10.1126/science.1261359

13. Deluca A, Corral Á. Fitting and goodness-of-fit test of non-truncated and truncated power-law distributions. Acta Geophys. 2013;61: 1351–1394. doi:10.2478/s11600-013-0154-9

14. Huttenhower C, Gevers D, Knight R, Abubucker S, Badger JH, Chinwalla AT, et al. Structure, function and diversity of the healthy human microbiome. Nature. Nature Publishing Group; 2012;486: 207–214. doi:10.1038/nature11234

15. Redford AJ, Bowers RM, Knight R, Linhart Y, Fierer N. The ecology of the phyllosphere: geographic and phylogenetic variability in the distribution of bacteria on tree leaves. Environ Microbiol. 2010;12: 2885–2893. doi:10.1111/j.1462-2920.2010.02258.x
